# miRNA-Guided Imaging and Photodynamic Therapy Treatment of Cancer Cells Using Zn(II)-Protoporphyrin IX-Loaded Metal–Organic Framework Nanoparticles

**DOI:** 10.1021/acsnano.1c04681

**Published:** 2022-01-12

**Authors:** Pu Zhang, Yu Ouyang, Yang Sung Sohn, Michael Fadeev, Ola Karmi, Rachel Nechushtai, Ilan Stein, Eli Pikarsky, Itamar Willner

**Affiliations:** † Institute of Chemistry, Center for Nanoscience and Nanotechnology, The Hebrew University of Jerusalem, Jerusalem 91904, Israel; ‡ Institute of Life Science, The Hebrew University of Jerusalem, Jerusalem 91904, Israel; § The Lautenberg Center for Immunology and Cancer Research, IMRIC, The Hebrew University of Jerusalem, Jerusalem 91120, Israel

**Keywords:** fluorescence, G-quadruplexes, hybridization chain reaction, breast cancer, ovarian cancer, reactive oxygen species

## Abstract

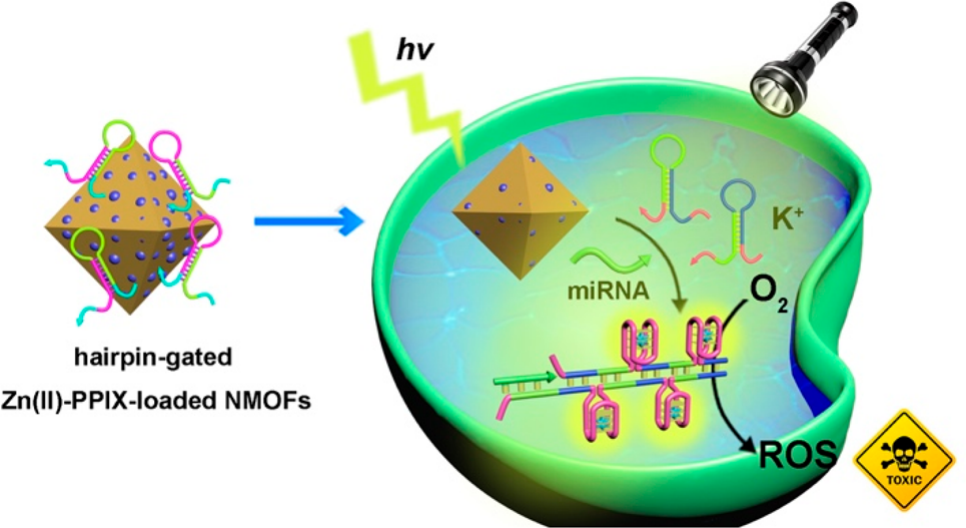

An analytical platform for the selective miRNA-21-guided imaging of breast cancer cells and miRNA-221-guided imaging of ovarian cancer cells and the selective photodynamic therapy (PDT) of these cancer cells is introduced. The method is based on Zn(II)-protoporphyrin IX, Zn(II)-PPIX-loaded UiO-66 metal–organic framework nanoparticles, NMOFs, gated by two hairpins H_i_/H_j_ through ligation of their phosphate residues to the vacant Zr^4+^-ions associated with the NMOFs. The hairpins are engineered to include the miRNA recognition sequence in the stem domain of H_i_, and in the H_i_ and H_j_, partial locked stem regions of G-quadruplex subunits. Intracellular phosphate-ions displace the hairpins, resulting in the release of the Zn(II)-PPIX and intracellular miRNAs open H_i_, and this triggers the autonomous cross-opening of H_i_ and H_j_. This activates the interhairpin hybridization chain reaction and leads to the assembly of highly fluorescent Zn(II)-PPIX-loaded G-quadruplex chains. The miRNA-guided fluorescent chains allow selective imaging of cancer cells. Moreover, PDT with visible light selectively kills cancer cells and tumor cells through the formation of toxic reactive oxygen species.

MicroRNAs, miRNAs, are short noncoding RNA sequences that regulate gene expression in multiple cellular adaptations.^1−3^ Up-regulation or down-regulation of miRNAs has been related to numerous biological processes, for example, cell proliferation,^4^ cell aging,^5^ apoptosis,^6^ and different diseases.^7^ Particularly, the identification of miRNAs related to different malignant cells finds growing interest, and miRNAs are important biomarkers for diagnosis, prognosis, progression, and recurrence of cancer. Different analytical methods to detect miRNAs were developed, including electrochemical,^8,9^ optical,^10,11^ and nanoparticle-based platforms.^12−15^ The low levels of intracellular miRNAs required, however, the development of amplified miRNA detection platforms. Different *in vitro* amplified miRNA detection schemes were reported, for example, polymerase-mediated rolling circle amplification,^16−18^ exponential exonuclease- or nuclease-assisted amplified detection of miRNAs,^19,20^ and DNAzyme-catalyzed analysis of miRNAs.^21^ Also, enzyme-free amplified sensing platforms of miRNAs were demonstrated, including the hybridization chain reaction (HCR),^22^ catalytic DNA hairpin assembly,^23^ and photoactivated toehold-mediated strand displacement process.^24^ In addition, the multiplexed analyses of miRNAs, multifunctional DNA nanostructures,^25,26^ and miRNA arrays,^27^ for parallel and high-throughput detection of the biomarkers, were reported.

The intracellular detection of miRNAs is particularly important for miRNAs imaging in cancer cells.^28^ Graphene oxide,^29^ polydopamine/ZnO nanoparticles,^30^ and carbon nitride^31,32^ were used as carriers for nucleic acid activating the intracellular HCR visualizing miRNAs. Similarly, an elegant miRNA-triggered concatenated HCR using functional DNA hairpins^33^ and fluorophore-labeled DNA hairpins-loaded Au nanoparticles for imaging intracellular miRNAs^34,35^ was demonstrated. miRNAs-triggered release of hairpins and catalytic hairpin assembly regenerating the miRNAs provided a useful method for the amplified imaging of miRNA-containing cells.^36,37^ Also, the collective intracellular imaging of miRNAs, the spatiotemporal fluorescence and electrochemical detection of miRNA, at single cell level was demonstrated.^38^

Moreover, miRNAs find growing interest as functional units for specific release of drugs from miRNA-responsive nano- or microcarriers.^39^ For example, drug-loaded DNA-gated mesoporous SiO_2_ nanoparticles were unlocked by miRNA, resulting in the release of drugs.^40^ Similarly, drug-loaded DNA-gated metal–organic framework nanoparticles, NMOFs, allowed the targeted miRNA-triggered and amplified release of drugs by the regeneration of miRNAs.^41^ Interestingly, multiplexed and selective release of drugs by different miRNAs, using a mixture of miRNA-responsive NMOFs, was demonstrated.

Recent advances in DNA nanotechnology addressed the development of nano- or microcarriers for theranostic applications. DNA-responsive carriers functionalized with gating units, such as pH or aptamer-ligand triggered locks were reported.^42^ For example, the development of “artificial pancreas” microcapsules^43^ or NMOFs^44^ releasing insulin, in response to glucose and pH triggers, or release of drugs from VEGF-responsive microcapsules,^45^ were demonstrated.

This discussion calls for the need to develop miRNA-responsive nanoparticles for theranostic applications and particularly functional nanoparticles for theranostic cancer cell imaging and therapeutic treatment. The present study will make use of NMOFs as functional carriers to protect DNA from nuclease degradation and enhance cellular uptake of the loads for theranostic applications. NMOFs find growing interest for sensing^46−48^ and drug delivery applications,^49−52^ and specifically, stimuli-responsive nucleic acid-modified NMOFs for triggered drug delivery were developed.^53,54^ These included pH-responsive i-motif or triplex gates^55^ and K^+^-ions/crown ether-responsive G-quadruplex locks^56^ or DNAzyme-gated NMOFs,^57^ and the hybrid systems were used for the switchable release of drugs. Furthermore, nucleic acid structures reconfigure, in the presence of appropriate triggers, into structures that bind auxiliary ligands to yield functional supramolecular assemblies revealing catalytic or optical properties.^58^ For example, pre-engineered DNA hairpins act as functional scaffolds for DNA-triggered HCR that yields catalytic G-quadruplex wires.^59^ Also, G-quadruplex structures^60^ were reported to bind Zn(II)-protoporphyrin IX, Zn(II)-PPIX, to yield highly fluorescent supramolecular assemblies. In addition, photodynamic therapies (PDT),^61−64^ and particularly porphyrin-photosensitized PDT treatment,^65^ attract growing interest for cancer therapy. Thus, the superior photophysical fluorescence functions of Zn(II)-PPIX bound to G-quadruplex chains could be used for effective photosensitized generation of reactive oxygen species (ROS) for PDT treatment. Accordingly, the porous high-loading capacities and biocompatibility of NMOFs and the properties of nucleic acid scaffolds provide a rich arsenal of molecular and material tools for developing hybrid theranostic systems.

Here, we report on the use of Zn(II)-PPIX-loaded nucleic acid-modified NMOFs as functional carriers for miRNA-guided imaging of cancer cells and for concomitant PDT of malignant cells. We demonstrate that the modification of Zn(II)-PPIX-loaded NMOFs with two hairpins leads to two complementary functions: (i) Cellular phosphate levels desorb the hairpin units from the NMOFs, resulting in the release of the Zn(II)-PPIX loads. As one of the desorbed hairpins includes a sequence that recognizes the miRNA specific for corresponding malignant cells, and since the hairpins are engineered to include G-quadruplex subunits and to induce the interhairpin HCR, the miRNA-triggered HCR between the hairpins generates G-quadruplex wires. The binding of released Zn(II)-PPIX to G-quadruplex units results in highly fluorescent Zn(II)-PPIX/G-quadruplex wires that enable the selective imaging of the cancer cells. (ii) The miRNA-guided HCR-stimulated formation of Zn(II)-PPIX/G-quadruplex wires yields effective photosensitizers for light-induced generation of ROS that lead to effective and selective PDT of respective malignant cells. Significantly, the formation of Zn(II)-PPIX/G-quadruplex chain is selectively guided by specific miRNAs present in the cancer cells, and thus, miRNAs guide the selective imaging and PDT treatment of the respective cancer cells (miRNA-21 in breast cancer cells,^66^ miRNA-221 in ovarian cancer cells^67^).

## Results and Discussion

**Figure 1:**
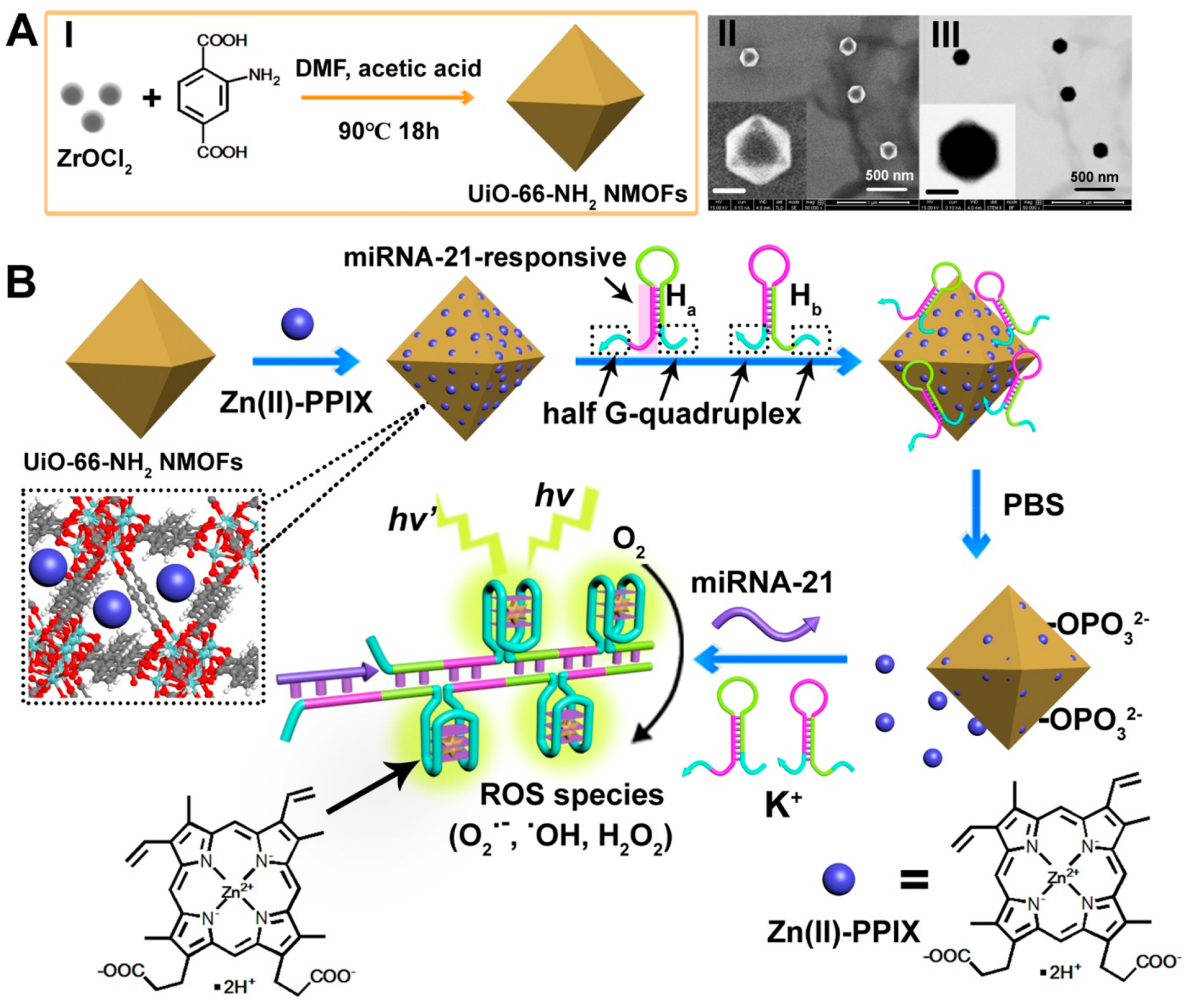
(A) (I) Synthesis, (II) SEM image, and (III) STEM image of UiO-66-NH_2_ NMOFs. The insets in panels II and III correspond to the magnified SEM and STEM images of UiO-66-NH_2_ NMOFs, respectively. Scale bar of insets is 100 nm. (B) Scheme for the loading of NMOFs with Zn(II)-PPIX photosensitizer and their gating by hairpins H_a_ and H_b_. The bound hairpins are displaced by phosphate-ions, resulting in the release of load and the miRNA-21-induced activation of HCR leading, in the presence of K^+^-ions, to the self-assembly of G-quadruplex chains that associate the Zn(II)-PPIX.

Amino-modified UiO-66 NMOFs, UiO-66-NH_2_ NMOFs, were synthesized by the reaction of ZrOCl_2_ with amino-terphthalic acid ligand (**1**),^68^Figure 1A(I). Figure 1A(II, III), shows the scanning electron microscopy (SEM) and transmission electron microscopy (TEM) images of bipyramidal 200 nm-sized NMOFs. The UiO-66-NH_2_ NMOFs were loaded with Zn(II)-PPIX and then gated with nucleic acid hairpins H_a_ and H_b_ that bind through the phosphate units of nucleic acids to the vacant ligation sites of Zr^4+^-ions of NMOFs, miRNA-21-responsive Zn(II)-PPIX-loaded H_a_/H_b_-locked NMOFs (Figure 1B). The loading of Zn(II)-PPIX within the UiO-66-NH_2_ NMOFs was further characterized by Brunauer–Emmett–Teller (BET) surface area analysis and pore volume analysis of the NMOFs before and after loading with the Zn(II)-PPIX (Table S1). While the pore volume of the NMOFs prior to loading corresponded to 0.901 cc/g, after loading with Zn(II)-PPIX, it decreased and corresponded to 0.647 cc/g. The surface area of the NMOFs prior the loading with Zn(II)-PPIX corresponded to 1641.750 m^2^/g, whereas after loading, it decreased to 1167.953 m^2^/g. These results are consistent with the binding of Zn(II)-PPIX to the pores. (For further characterization of the NMOFs, see Figure S1.) It should be noted that the morphology of the UiO-66-NH_2_ NMOFs is unchanged upon loading the particles with Zn(II)-PPIX and the gating of the loaded NMOFs with H_a_ and H_b_ (Figure S2). The loading of H_a_/H_b_ on UiO-66-NH_2_ NMOFs was evaluated spectroscopically to be 32 nmole·mg^–1^ of particles (Figure S3). It should be noted that the amine-terphtalic acid ligand was selected to bridge Zr^4+^-ions and preferred over the unsubstituted teterphthalic ligand, since we find that the loading of H_a_ and H_b_ on UiO-66-NH_2_ NMOFs is ca. 2-fold higher as compared to unsubstituted UiO-66 NMOFs, Figure S3. The mechanism to unlock Zn(II)-PPIX loads and to apply them for imaging and PDT treatment of cancer cells is depicted in Figure 1B. At high intracellular phosphate concentration, or in the presence of phosphate buffer saline (PBS), ligand exchange of the phosphate units associated with the hairpin nucleic acids, H_a_ and H_b_ bound to the NMOFs, by the phosphate ions in solution proceeds. The ligand exchange is driven by the high concentration of phosphate ions in solution and is dominated by dynamic equilibrium of the phosphate unit linked to the Zr^4+^-vacant sites. H_a_ and H_b_ are dissociated from NMOFs, leading to the release of the Zn(II)-PPIX which is accommodated in interpore domains of the NMOFs. The hairpin H_a_ is pre-engineered to include the recognition sequence for miRNA-21 in its stem domain. In addition, hairpins H_a_ and H_b_ are pre-engineered to include the sequences to induce the interhairpin HCR process upon miRNA-21 triggered opening of hairpin H_a_ and G-quadruplex sequences capable to self-assemble in G-quadruplex upon the formation of the HCR biopolymer. The G-quadruplex subunits exist in a partial locked configuration associated with the hairpin stem domains extended by single-strand tethers. Thus, the miRNA-21-stimulated opening of H_a_ and H_b_ leads, in the presence of K^+^-ions (at cellular concentrations of ca. 125 mM),^69,70^ to the formation of HCR wires that include tethered G-quadruplex units. The association of released Zn(II)-PPIX to G-quadruplex units is known to yield highly fluorescent Zn(II)-PPIX/G-quadruplex components, and these are planned to act as intracellular imaging constituents. In addition, the superior photophysical properties of the Zn(II)-PPIX G-quadruplex wires (located inside the cells) are anticipated to yield intracellular ROS for PDT treatment of the malignant cells upon irradiation, selectively in miRNA-21-overexpressed breast cancer cells. For further circular dichroism (CD) and time-resolved experiments supporting the selective interaction of Zn(II)-PPIX with G-quadruplex structures and demonstrating the superior photophysical properties of Zn(II)-PPIX bound to the G-quadruplex structure, see Figures S4 and S5.

**Figure 2:**
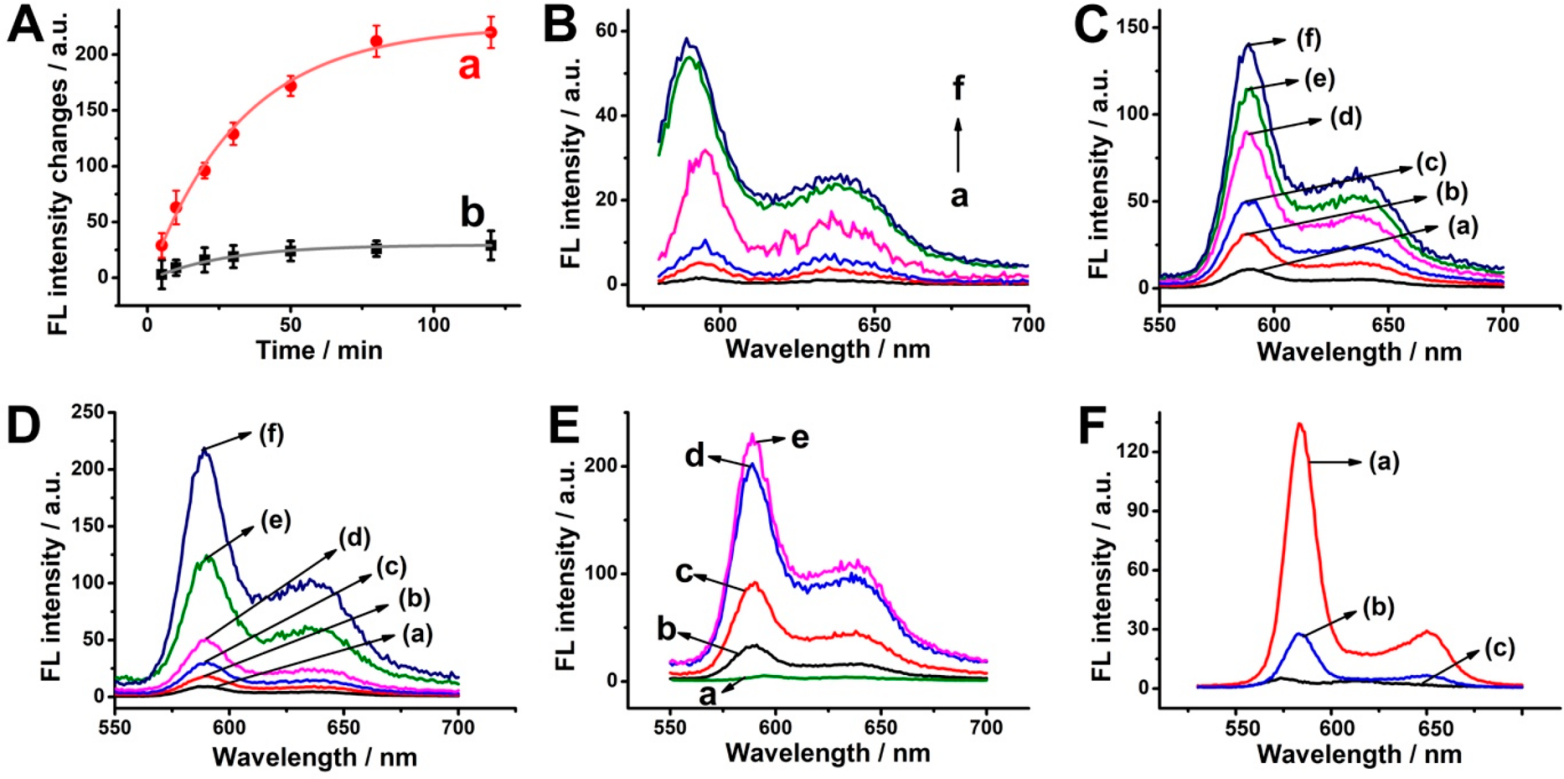
(A) Time-dependent fluorescence changes of Zn(II)-PPIX/G-quadruplex chains generated by miRNA-21-responsive Zn(II)-PPIX-loaded H_a_/H_b_-locked NMOFs treated with (a) PBS, (b) HEPES buffer. 0.1 mg NMOFs are treated with 100 μL of PBS or HEPES buffer, 10 mM, miRNA-21, 200 nM, and K^+^-ions, 50 mM. (B) Fluorescence spectra of Zn(II)-PPIX/G-quadruplex chains generated after a fixed time-interval of 15 min using variable concentrations of K^+^: (a) 0 mM K^+^, (b) 10 mM K^+^, (c) 20 mM K^+^, (d) 50 mM K^+^, (e) 100 mM K^+^, (f) 140 mM K^+^. 0.1 mg NMOFs are treated with 100 μL of PBS, 10 mM, miRNA-21, 200 nM. (C) Fluorescence spectra of Zn(II)-PPIX/G-quadruplex chains generated after a fixed time interval of 15 min using variable concentrations of miRNA-21: (a) 0 nM, (b) 100 nM, (c) 200 nM, (d) 500 nM, (e) 1 μM, (f) 2 μM. 0.1 mg NMOFs are treated with 100 μL of PBS, 10 mM, K^+^-ions, 50 mM. (D) Fluorescence spectra of Zn(II)-PPIX/G-quadruplex chains generated after a fixed time-interval of 15 min using variable concentrations of PBS: (a) 0 mM, (b) 5 mM, (c) 7.5 mM, (d) 10 mM, (e) 20 mM, (f) 50 mM. 0.1 mg NMOFs are treated with 100 μL of PBS, 10 mM, miRNA-21, 200 nM, and K^+^-ions, 50 mM. (E) Fluorescence spectra of Zn(II)-PPIX/G-quadruplex chains generated using different time intervals for operating the HCR process: (a) 0 min, (b) 15 min, (c) 30 min, (d) 60 min, (e) 120 min. 0.1 mg NMOFs are treated with 100 μL of PBS, 10 mM, miRNA-21, 200 nM, and K^+^-ions, 50 mM. (F) Fluorescence spectra of Zn(II)-PPIX/G-quadruplex chains generated using (a) miRNA-21, 200 nM; (b) miRNA-221, 200 nM; (c) miRNA-145, 200 nM. 0.1 mg NMOFs are treated with 100 μL of PBS, 10 mM, and K^+^-ions, 50 mM. Error bars derived from *N* = 3 experiments.

Figure 2A depicts the time-dependent fluorescence changes observed in the solution upon treatment of the miRNA-21-responsive Zn(II)-PPIX-loaded H_a_/H_b_-locked NMOFs with miRNA-21, 200 nM, K^+^-ions, 50 mM in PBS, 10 mM (a), and nonphosphate buffer, HEPES buffer, 10 mM (b). The fluorescence intensities of Zn(II)-PPIX increase with time, in PBS (a), while only minute fluorescence changes are observed in the HEPES solution (b). These results are consistent with the phosphate-induced release of H_a_/H_b_ and subsequent formation of miRNA-21-triggered fluorescent Zn(II)-PPIX/G-quadruplex wires. The lack of fluorescence change in the presence of HEPES buffer is due to the prohibited unlocking of NMOFs in the absence of phosphate-ions. Further control experiments revealed that in the absence of miRNA-21, only minute fluorescence changes are observed, since the HCR process is prohibited, yet low fluorescent Zn(II)-PPIX is released (Figure S6, (i)). We find that the fluorescence generated by the Zn(II)-PPIX/G-quadruplex wires is controlled by the concentrations of K^+^-ions (Figure 2B). While in the absence of K^+^-ions, no fluorescence is observed, elevating the concentration of K^+^-ions intensified the fluorescence of the Zn(II)-PPIX/G-quadruplex wires and the fluorescence levels off at a concentration of K^+^-ions corresponding to 100 mM. The time-dependent fluorescence changes of Zn(II)-PPIX/G-quadruplex wires reach a saturation value after ca. 80 min. The saturated fluorescence is observed upon the complete release of Zn(II)-PPIX that occupies G-quadruplex units in the HCR wires. Using an appropriate calibration curve of Zn(II)-PPIX in a G-quadruplex configuration, we estimate that ca. 80 nmoles·mg^–1^ of Zn(II)-PPIX were released from NMOFs, a value that agrees well with the loading degree of Zn(II)-PPIX in NMOFs (Figure S7). (The phosphate-induced release of Rhodamine 6G from H_a_/H_b_-gated NMOFs was further demonstrated (Figure S8).) Figure 2C depicts the fluorescence spectra of Zn(II)-PPIX/G-quadruplex chains generated upon treatment of loaded NMOFs in PBS with different concentrations of miRNA-21 for a fixed time-interval of 15 min. As the concentration of miRNA-21 increases, the fluorescence of resulting chains is intensified, consistent with the enhanced miRNA-induced opening of H_a_/H_b_, and the formation of Zn(II)-PPIX/G-quadruplex chains. Figure 2D shows the fluorescence spectra of the resulting Zn(II)-PPIX/G-quadruplex chains in the presence of 200 nM miRNA-21 and variable concentrations of phosphate for a fixed time interval of 15 min. As phosphate concentrations increase, the fluorescence of Zn(II)-PPIX chains is intensified, consistent with the increased phosphate-triggered release of H_a_/H_b_ that acts as the source for the miRNA-driven HCR process. Interestingly, in the absence of phosphate, no Zn(II)-PPIX/G-quadruplex chains are formed since the release of hairpins from the NMOFs is prohibited. Figure 2E depicts the fluorescence intensities of the Zn(II)-PPIX/G-quadruplex chains formed upon driving HCR for different time intervals in the presence of fixed concentrations of PBS and miRNA-21. As the time intervals of HCR are prolonged, the fluorescence intensities of Zn(II)-PPIX are elevated, consistent with the higher contents of the Zn(II)-PPIX/G-quadruplex fluorescence units. After ca. 80 min of the HCR process, the fluorescence intensities of resulting Zn(II)-PPIX/G-quadruplex chains reaches a constant saturation value, due to the depletion of hairpins (Figure 2E and Figure S9). Furthermore, the formation of Zn(II)-PPIX/G-quadruplex chains is selective and proceeds only with miRNA-21. Figure 2F shows the fluorescence spectra of Zn(II)-PPIX/G-quadruplex chains generated upon treatment of miRNA-21-responsive H_a_/H_b_-gated Zn(II)-PPIX-loaded NMOFs in PBS and in the presence of miRNA-21 (curve (a)), miRNA-221 (curve (b)), and miRNA-145 (curve (c)), respectively. Control experiments revealed that treatment of the miRNA-21-responsive NMOFs with miRNA-221 or miRNA-145 did not lead to the triggering of G-quadruplex chains, indicating that the generation of G-quadruplex chains from respective NMOFs is specific to miRNA-21. The selectivity originated from the fact that the hairpins H_a_ and H_b_ are engineered to be opened and stimulate the HCR process only in the presence of miRNA-21. The formation of the G-quadruplex chains triggered by different concentrations of miRNA-21 in the presence of pure buffer and in the presence of 10% serum solution was confirmed by electrophoretic separation (Figure S10). The G-quadruplex configuration embedded in the biopolymers was confirmed by CD (Figure S11). It should be noted that the phosphate-induced release of the hairpins H_a_ and H_b_ and of the Zn(II)-PPIX-load proceeds in parallel to stimulate the miRNA-21 triggered HCR generation of the Zn(II)-PPIX/G-quadruplex wires. While the Zn(II)-PPIX load can not diffuse out of the NMOFs in the locked hairpin configuration, the kinetics of the diffusional release of Zn(II)-PPIX upon the phosphate-ions-induced removal of the hairpins possibly could affect the HCR process, generating the Zn(II)-PPIX/G-quadruplex wires. To assess the contribution of the release rate of hairpins H_a_ and H_b_ and the release rate of Zn(II)-PPIX to the rate of the formation of photoactive wires, we performed several control experiments addressing this issue, and these are described in Figures S12 and S13. Based on these control experiments, we conclude that the rate of diffusional release of Zn(II)-PPIX has very little effect on the resulting HCR process and the miRNA-triggered formation of the Zn(II)-PPIX/G-quadruplex wires.

The result in Figure 2F suggests, however, that appropriate engineering of hairpins recognizing other miRNAs and the design of hairpins-modified NMOFs could yield other selective miRNA-responsive NMOFs. Indeed, the miRNA-guided HCR-stimulated generation of fluorescent Zn(II)-PPIX/G-quadruplex chains by the miRNA-221 that acts as specific biomarker for ovarian cancer cells was demonstrated (Figures S14–S16) and accompanying discussion.

**Figure 3:**
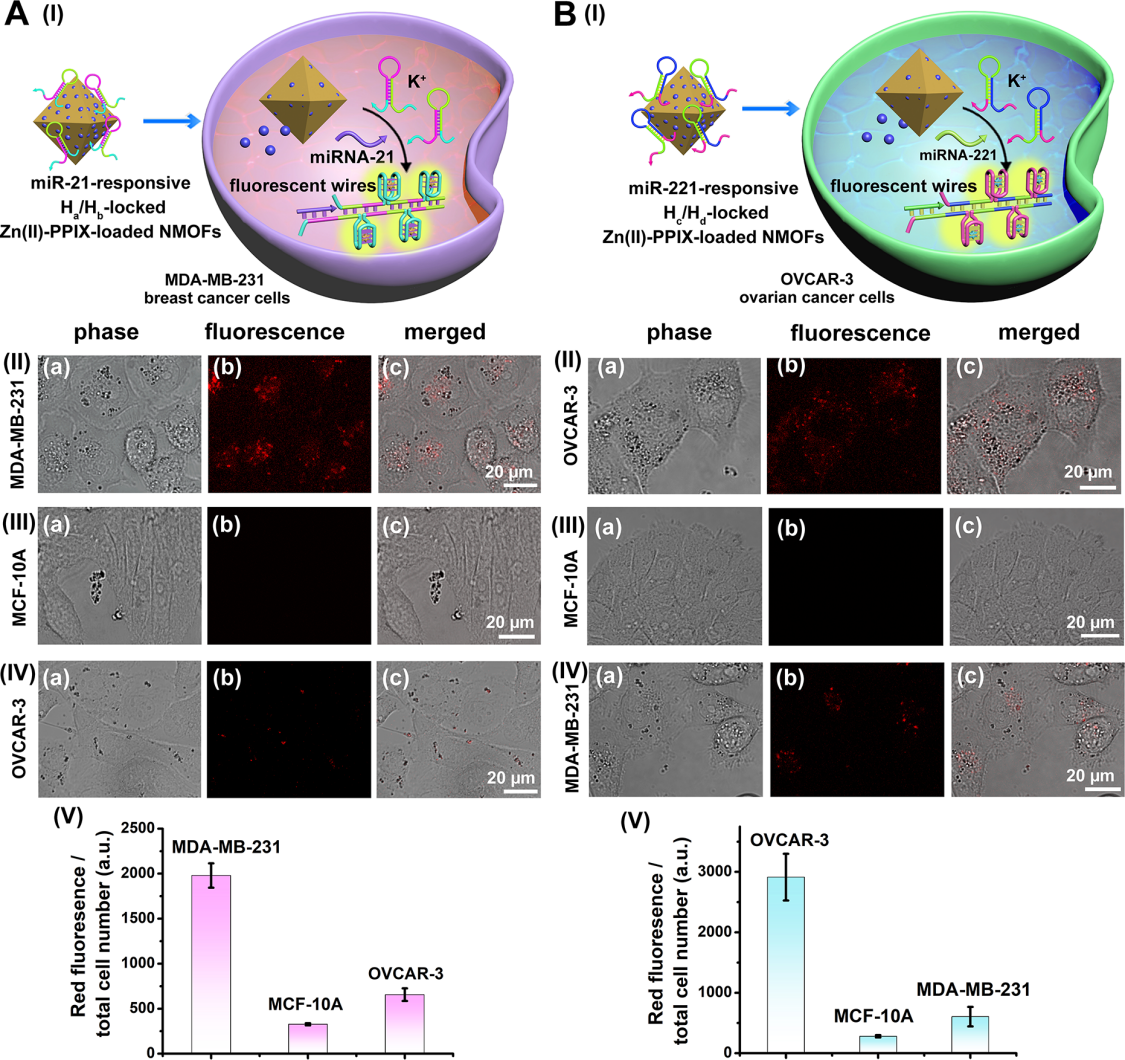
(A) (I) Schematic imaging of MDA-MB-231 cells that consist of overexpressed miRNA-21 using miRNA-21-responsive H_a_/H_b_-gated Zn(II)-PPIX-loaded NMOFs. Confocal microscopy images of: (II) MDA-MB-231 cells, (III) MCF-10A cells, and (IV) OVCAR-3 cells. (a) Bright-field confocal microscopy images. (b) Fluorescence confocal microscopy images of Zn(II)-PPIX/G-quadruplex wires generated in the cells. (c) Merged images. (V) Integrated normalized fluorescence intensities of different cells treated with miRNA-21-responsive NMOFs. (B) (I) Schematic imaging of OVCAR-3 cells that include overexpressed miRNA-221 using the miRNA-221-responsive H_c_/H_d_-gated Zn(II)-PPIX-loaded NMOFs. Confocal microscopy images of: (II)OVCAR-3 cells, (III) MCF-10A cells, and (IV) MDA-MB-231 cells. (a) Bright-field confocal microscopy images. (b) Fluorescence confocal microscopy images of Zn(II)-PPIX/G-quadruplex wires generated in respective cells. (c) Merged images. (V) Integrated normalized fluorescence intensities of these cells treated with the miRNA-221-responsive NMOFs. Error bars derived from *N* = 3 experiments.

Figure 3 shows the selective fluorescence imaging of miRNA-21 overexpressed MDA-MB-231 breast cancer cells and of miRNA-221 overexpressed OVCAR-3 ovarian cancer cells. In Figure 3A(I), the permeation features of the miRNA-21-responsive H_a_/H_b_-gated Zn(II)-PPIX-loaded NMOFs with the breast cancer cells, the intracellular phosphate-induced release of Zn(II)-PPIX from NMOFs, and the formation of overexpressed miRNA-21-triggered fluorescent Zn(II)-PPIX/G-quadruplex wires are schematically presented. The fluorescence confocal microscopy images of MDA-MB-231 cells, MCF-10A epithelial cells, and OVCAR-3 cells treated with miRNA-21-responsive NMOFs are shown in Figure 3A(II–IV). Strong fluorescence is observed in MDA-MB-231 cells, while significantly lower fluorescence is observed in MCF-10A cells or OVCAR-3 cells upon treatment with NMOFs. Figure 3A(V) shows normalized fluorescence intensities generated by the respective cells. The results indicated ca. 4-fold higher fluorescence generated in MDA-MB-231 cancer cells, as compared to substantially lower fluorescence intensities in control systems. The results are consistent with the selective miRNA-21-stimulated activation of the HCR process in phosphate-unlocked NMOFs that released Zn(II)-PPIX. The resulting fluorescent Zn(II)-PPIX/G-quadruplex wires led to the selective fluorescence in MDA-MB-231 cells. Similarly, the permeation features of miRNA-221-responsive H_c_/H_d_-gated Zn(II)-PPIX-loaded NMOFs with OVCAR-3 cells, the intracellular phosphate-induced release of Zn(II)-PPIX from NMOFs and the formation of overexpressed miRNA-221-triggered fluorescent Zn(II)-PPIX/G-quadruplex wires are schematically presented in Figure 3B(I). Figure 3B(II–IV) presents the fluorescence confocal microscopy images of OVCAR-3 cells, MCF-10A epithelial cells, and MDA-MB-231 cells treated with the miRNA-221-responsive NMOFs. Figure 3B(V) shows the normalized fluorescence intensities generated by different cells subjected to the NMOFs. Evidently, the ovarian cancer cells show high fluorescence intensities, whereas the other types reveal substantial lower fluorescence intensities. The results are consistent with selective miRNA-221-induced activation of HCR process of phosphate-stimulated release of H_c_/H_d_ associated with NMOFs and the release of the Zn(II)-PPIX. The miRNA-221 triggered formation of highly fluorescent Zn(II)-PPIX/G-quadruplex wires provides an opportunity for selective imaging of ovarian cancer cells. The results demonstrate the selective miRNA-guided imaging of the respective cancer cells and the differentiation from nonmalignant epithelial cells, consistent with the lack or low content of specific miRNAs in the corresponding cells. Compared to the naked hairpin/Zn(II)-PPIX solution, cellular uptake of hairpin-gated Zn(II)-PPIX-loaded NMOFs was significantly enhanced (Figure S17), indicating that the NMOFs protect hairpins from nuclease degradation and enhance cellular uptake of the loads, facilitating the internalization via endocytosis pathways.

**Figure 4:**
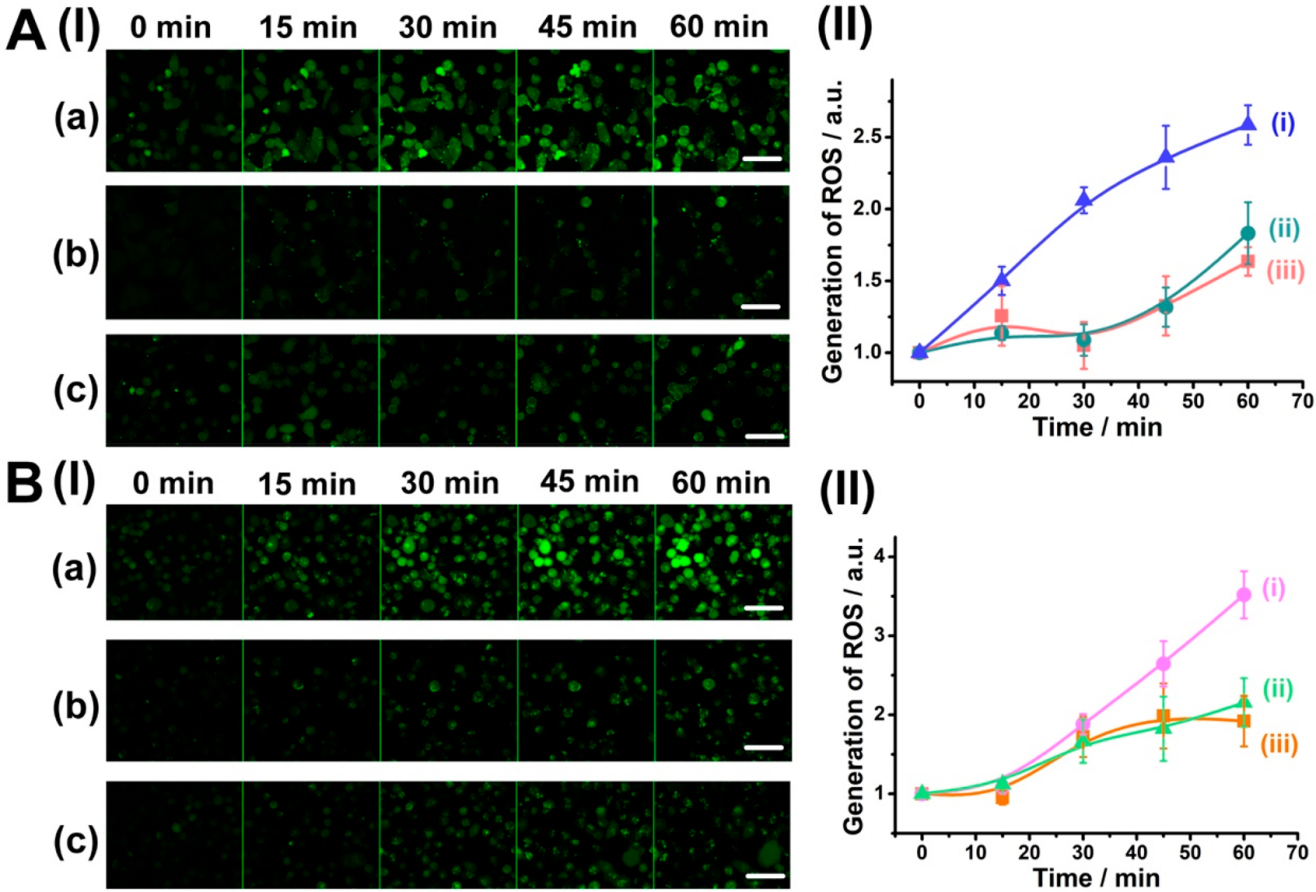
(A) (I) Time-dependent fluorescence images of ROS indicator in MDA-MB-231 cells: (a) treated with miRNA-21-responsive NMOFs, (b) treated with miRNA-221-responsive NMOFs, (c) untreated cells. Scale bars: 100 μm. (II) Normalized time-dependent fluorescence intensities of ROS indicator in MDA-MB-231 cells corresponding to (i) treated with miRNA-21-responsive NMOFs, (ii) treated with miRNA-221-responsive NMOFs, (iii) untreated cells. (B) (I) Time-dependent fluorescence images of ROS indicator in OVCAR-3 cells: (a) treated with miRNA-221-responsive NMOFs, (b) treated with miRNA-21-responsive NMOFs, (c) untreated cells. Scale bars: 100 μm. (II) Normalized time-dependent fluorescence intensities of ROS indicator in OVCAR-3 cells corresponding to (i) treated with miRNA-221-responsive NMOFs, (ii) treated with miRNA-21-responsive NMOFs, (iii) untreated cells. Error bars derived from *N* = 3 experiments.

The high fluorescent properties of Zn(II)-PPIX/G-quadruplex chains triggered by specific miRNAs suggest that these nanostructures could act as effective photosensitizers for light-induced formation of ROS for the selective PDT treatment of malignant cells. The photodynamic efficacy of *in vitro* generated Zn(II)-PPIX/G-quadruplex chains was determined by measuring ROS generation using 1,3-diphenylisobenzofuran (DPBF) as an indicator (Figure S18). In addition, the specific formation of the ROS intermediates O_2_^•–^, H_2_O_2_ and OH• upon irradiation of the Zn(II)-PPIX/G-quadruplex chains was confirmed using chemiluminescence (O_2_^•–^), electron spin resonance (ESR) spectroscopy (OH•), and H_2_O_2_ enzymatic assay (Figure S19). In the next step, the selective intracellular formation of ROS species in two types of cancer cells, including miRNA-21 overexpressing MDA-MB-231 breast cancer cells (Figure 4A) and miRNA-221 overexpressing OVCAR-3 ovarian cancer cells (Figure 4B), was demonstrated. The cells were treated with ROS imaging fluorescent probe, di(acetoxymethyl ester)-6-carboxy-2′,7′-dichlorodihydrofluorescein diacetate (CDCHF-DA) and were subjected to visible light illumination, λ = 532 nm for 12 min, 40 mW/cm^2^. The time-dependent fluorescent changes in cells were followed. The fluorescence of the ROS probe is intensified with time in the MDA-MB-231 cells treated with miRNA-21-responsive NMOFs, Figure 4(a), while very weak fluorescence is developed in MDA-MB-231 cells treated with miRNA-221-responsive NMOFs and the untreated MDA-MB-231 cells. Figure 4A(II) shows the time-dependent normalized fluorescence changes of ROS imaging probe in the MDA-MB-231 cells: (i) treated with miRNA-21-responsive NMOFs, (ii) treated with miRNA-221-responsive NMOFs, and (iii) untreated cells. A clear increase in the fluorescence of ROS indicator in the MDA-MB-231 cells treated with miRNA-21-responsive NMOFs is observed. These results are consistent with the fact that miRNA-21 is overexpressed in MDA-MB-231 breast cancer cells, while miRNA-221 is low in MDA-MB-231 cells. As a result, only miRNA-21-responsive NMOFs lead to effective formation of Zn(II)-PPIX/G-quadruplex photosensitizer chains in MDA-MB-231 cells, resulting in selective effective photosensitized generation of ROS species in these cells. Similarly, the fluorescence of ROS indicator is intensified with time in the ovarian cells treated with miRNA-221-responsive NMOFs, Figure 4(a), while only weak fluorescence is observed in the ovarian cells treated with miRNA-21-responsive NMOFs and untreated cells, Figure 4(b,c). Figure 4B(II) depicts the time-dependent integrated fluorescence intensities in the ovarian cancer cells: (i) treated with miRNA-221-responsive NMOFs, (ii) treated with miRNA-21-responsive NMOFs, and (iii) untreated cells. Intense fluorescence of the ROS probe indicator is observed in ovarian cancer cells with miRNA-221-responsive NMOFs, while substantially lower fluorescence intensities were observed for the OVCAR-3 cells treated with miRNA-21-responsive NMOFs and untreated cells. These results agree well with the fact that miRNA-221 is overexpressed in ovarian cancer cells, while breast cancer cells include a low amount of miRNA-21. As a result, miRNA-221-guided formation of Zn(II)-PPIX/G-quadruplex photosensitizer chains proceeds in OVCAR-3 ovarian cancer cells, leading to the effective photosensitized generation of ROS species in these cells. Thus, our results demonstrated selective generation of ROS species in respective malignant cells by the respective stimuli-responsive NMOFs.

**Figure 5:**
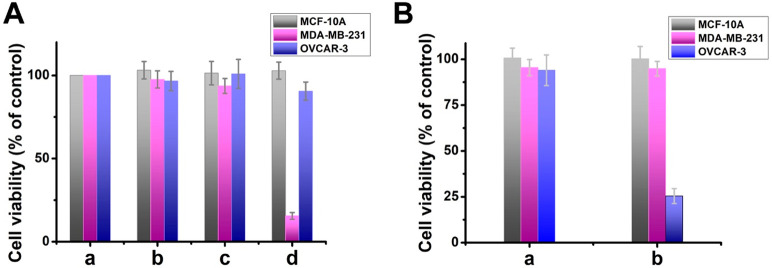
Cell viability of PDT-treated cells: MCF-10A, MDA-MB-231, OVCAR-3, and control systems. (A) (a) Untreated cells. (b) Cells treated with light in the absence of NMOFs. (c) Cells treated with miRNA-21-responsive NMOFs without light. (d) Cells treated with the miRNA-21-responsive NMOFs and light. (B) (a) Cells treated with the miRNA-221-responsive NMOFs without light. (b) Cells treated with the miRNA-221-responsive NMOFs and light. Error bars derived from *N* = 3 experiments. (*p* < 0.0001).

The selective miRNA-induced formation of ROS intermediates was then applied to evaluate PDT-stimulated cytotoxicity toward the MDA-MB-231 breast cancer cells, OVCAR-3 ovarian cancer cells, and MCF-10A epithelial breast cells. Figure 5A shows the cell viabilities upon PDT of the cells with miRNA-21-responsive H_a_/H_b_-gated Zn(II)-PPIX-loaded NMOFs and applying appropriate control experiments. Figure 5A(a) corresponds to untreated cells. The irradiation of untreated cells did not alter cell growth kinetics for a time interval of 3 days, and Figure 5A(b) demonstrates that the irradiation is not toxic to the cells. In the absence of irradiation, the miRNA-21-responsive NMOFs have no cytotoxic effect on all three types of cells (Figure 5A(c)). Figure 5A(d) shows the respective cell viabilities, subjected to miRNA-21-responsive NMOFs and irradiated a continuous wave (CW) laser source, λ = 532 nm, 40 mW/cm^2^, for 12 min. An impressive cell death (85%) is observed for MDA-MB-231 cells, while no significant effect is observed for MCF-10A or OVCAR-3 cells. These results are consistent with the fact that in the miRNA-21-containing MDA-MB-231 cells, the effective formation of Zn(II)-PPIX/G-quadruplex photosensitizer chains proceeds, due to the intracellular phosphate-induced dissociation of H_a_/H_b_ locking units and the release of load. The low cytotoxicity effect of the PDT treatment toward MCF-10A or OVCAR-3 is due to the lack or low content of miRNA-21 in these cells. To rule out the possibility that MDA-MB-231 may be inherently more sensitive to PDT than OVCAR-3 and MCF-10A cells, we performed the converse experiment with NMOFs that are triggered by miRNA-221. Figure 5B depicts the cell viabilities of OVCAR-3 cells, MCF-10A cells, and MDA-MB-231 cells upon incubation with miRNA-221-responsive NMOFs. In the absence of irradiation, the NMOFs have no cytotoxic effect on all three types of cells (Figure 5B(a)). The NMOFs-treated cells were irradiated by visible light irradiation at λ = 532 nm, 40 mW/cm^2^ for 12 min, and after a time-interval of 3 days, and the cytotoxicity effect is shown in Figure 5B(b). A minute cytotoxic effect is observed toward the MCF-10A cells and MDA-MB-231 cells, yet significant ovarian cancer cell death is observed (ca. 75%). These results are consistent with the selective miRNA-221-guided PDT-stimulated cytotoxicity toward the ovarian cancer cells that are overexpressed with miRNA-221 and the lack of cytotoxic effect toward the MDA-MB-231 malignant cells or the MCF-10A epithelial cells that have low amounts of this biomarker. It should be noted that previous reports applied photosensitizer/G-quadruplex structures associated with pH-responsive polymers^71^ or nucleic acid-functionalized Au nanoparticles^72^ for intracellular release of PDT active agents. Beyond the introduction of an alternative PDT agent NMOFs carriers, our hairpin-gated Zn(II)-PPIX-loaded NMOFs reveal important selectivity and programmable features. The fact that our carriers are unlocked by specific miRNA biomarkers suggest that the active PDT agent will be generated only in the biomarker-containing cancer cells. In addition, the engineering of the hairpin gating units provides versatile means to design active PDT agents for target cancer cells.

**Figure 6:**
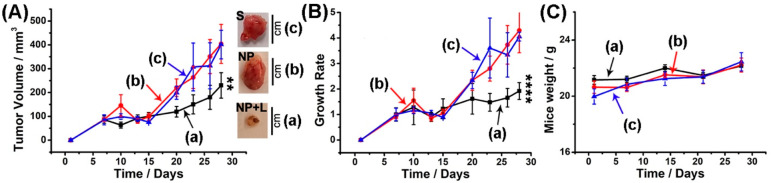
(A) Tumor volume profiles, (B) growth rate of the tumors, and (C) the corresponding body weight changes of xenograft epithelial MDA-MB-231 breast cancer tumors bearing NOD-SCID mice that were treated with (a) photoirradiated H_a_/H_b_-gated Zn(II)-PPIX-loaded NMOFs, NP+L, (b) photoirradiated H_a_/H_b_-gated NMOFs lacking Zn(II)-PPIX, NP, and (c) salines.

Preliminary *in vivo* experiments were performed by following the PDT treatment on MDA-MB-231 breast cancer tumors developed in mice (Figure 6). In these experiments, xenograft epithelial MDA-MB-231 breast cancer tumors were developed in NOD-SCID mice, and these were subjected to PDT using H_a_/H_b_-gated Zn(II)-PPIX-loaded NMOFs. As controls, NOD-SCID mice carrying the MDA-MB-231 tumors were subjected to H_a_/H_b_-gated NMOFs lacking the Zn(II)-PPIX load upon irradiation, and non-PDT treated mice injected with saline were evaluated. The PDT involved irradiation with a CW laser, 532 nm, 40 mW/cm^2^ for a time-interval of 15 min, for each PDT treatment, along a period of 30 days applying a total of 7 injections at a time-interval of 2–3 days. Figure 6A depicts the average time-dependent volume changes of the tumors in the different mice samples. While a small tumor volume change is observed for the PDT treated mice with the H_a_/H_b_-gated Zn(II)-PPIX-loaded NMOFs, ca. 200 mm^3^ after 30 days, Figure 6A(a), the mice treated with the H_a_/H_b_-gated, photosensitizer absent, NMOFs, or the untreated mice developed substantially larger tumors, ca. 400 mm^3^ after 30 days. These results imply that the vacant H_a_/H_b_-gated NMOFs are nontoxic toward the mice and that the PDT of the mice with the H_a_/H_b_-gated Zn(II)-PPIX-loaded NMOFs resulted in an inhibition in the tumor growth. Similarly, the growth rates of the tumors (width^2^ × height/2) in Figure 6B reveal similar conclusions, demonstrating an obvious inhibition in the growth rate of the tumors in the PDT treated mice using the H_a_/H_b_-gated Zn(II)-PPIX-loaded NMOFs, Figure 6B(a) *vs* (b) and (c). Finally, Figure 6C depicts the average weight of the mice along the time duration of the experiment. No weight loses are observed indicating that the NMOFs, and particularly the H_a_/H_b_-gated Zn(II)-PPIX-loaded NMOFs, are nontoxic toward the mice.

## Conclusions

The present study has introduced a versatile platform for the selective miRNA-guided fluorescence imaging and PDT treatment of cancer cells. UiO-66-NH_2_ NMOFs were loaded with Zn(II)-PPIX and gated by two hairpins H_i_/H_j_ ligated to the vacant Zr^4+^-ions sites on NMOFs by phosphate units associated with the DNA backbone. The hairpins H_i_/H_j_ were engineered to include in the stem region of H_i_, the miRNA recognition sequence, and in their partial locked stem domains caged G-quadruplex units. Intracellular phosphate-ions displaced the hairpin units and released the Zn(II)-PPIX. The intracellular miRNA-triggered opening of hairpin H_i_ and the subsequent activation of the interhairpin HCR yielded highly fluorescent Zn(II)-PPIX-loaded G-quadruplex chains. These enabled selective, miRNA-guided imaging of cancer cells and selective PDT of cancer cells through the generation of ROS. The selective imaging and PDT of MDA-MB-231 breast cancer cells and of OVCAR-3 ovarian cancer cells was demonstrated using the respective miRNA-21 and miRNA-221 as biomarkers. In principle, the concept can be extended to image and PDT treatment of any other diseased cells containing a characteristic overexpressed miRNA. While the present imaging and PDT therapeutic processes were based on the hairpins engineered toward the respective miRNA, one may envisage the broadening of the concept by engineering into the hairpin domains other recognition sequences, such as biomarker specific aptamers. The application of the platform for *in vivo* treatment of ovarian cancer, accompanied by histological evaluation of the treated tissues, is underway in our laboratories.

## Experimental Section

### miRNA-21-Responsive H_a_/H_b_-Gated Zn(II)-PPIX-Loaded NMOFs and the Release of the Zn(II)-PPIX

The miRNA-21-responsive H_a_/H_b_-gated Zn(II)-PPIX-loaded NMOFs, 0.1 mg, were subjected to 1 mL of respective buffer solutions (PBS or control buffer, HEPES buffer). At appropriate time intervals, samples of the mixture are centrifuged to precipitate the NMOFs (10,000 rpm for 2 min). Different concentrations of miRNA-21 and 50 mM K^+^ were added to the supernatant solution and incubated at room temperature for 3 h to generate the Zn(II)-PPIX/G-quadruplex photosensitizer chains. The fluorescence of the chains in the supernatant solution was measured using a Cary Eclipse fluorescence spectrophotometer (Varian Inc.).

### Confocal Microscopy Measurements

Cells, 2 × 10^5^, were planted in μ-slide 4-well with glass bottom on 1 day prior to the experiment. Cells were incubated with miRNA-21-responsive Zn(II)-PPIX-loaded H_a_/H_b_-locked NMOFs, 60 μg/mL, or miRNA-221-responsive H_c_/H_d_-gated Zn(II)-PPIX-loaded NMOFs, 60 μg/mL, for 6 h, then washed with DMEM-HEPES twice, and exposed to visible light irradiation, λ = 532 nm for 12 min, 30 mW/cm^2^. Red fluorescence in cells was monitored with the confocal microscopy (the Olympus FV3000 confocal laser-scanning microscope), and all images were analyzed with image J.

### ROS Production

ROS production in cancer cells was determined by incubating cells, 2 × 10^5^, loaded with miRNA-21-responsive Zn(II)-PPIX-loaded H_a_/H_b_-locked NMOFs, 60 μg/mL, or miRNA-221-responsive H_c_/H_d_-gated Zn(II)-PPIX-loaded NMOFs, 60 μg/mL, at 37 °C with 10 μM di(acetoxymethyl ester)-6-carboxy-2′,7′-dichlorodihydrofluorescein diacetate (CDCHF-DA) in HEPES-buffered saline (HBS) supplemented with 10 mM glucose after the exposure of cells to the visible light (λ = 532 nm for 12 min, 40 mW/cm^2^). This nonfluorescent molecule is readily converted to a green-fluorescent form when the acetate groups are removed by intracellular esterases and oxidation by the activity of ROS within the cells. The conversion of the nonfluorescent indicator to the green fluorescent indicator was measured on line for 1 h at 37 °C under the confocal microscopy (the Olympus FV3000 confocal laser-scanning microscope) (λ_ex_ = 488 nm; λ_em_ = 517 nm), and all images were analyzed with image J.

## Supporting Information Available

Materials and instrumentation, preparation of the loaded DNA NMOFs, release of the NMOFs, the cell experiment, and additional results PDF)
