# Supplementary material for: miRNA-Guided Imaging and Photodynamic Therapy Treatment of Cancer Cells Using Zn(II)-Protoporphyrin IX-Loaded Metal–Organic Framework Nanoparticles
Source: ACS Nano. 2022 Jan 12;16(2):1791–801. doi: 10.1021/acsnano.1c04681 (PMC8867907; doi:10.1021/acsnano.1c04681)
Supplement: Supplementary file 1 — nn1c04681_si_001.pdf [file nn1c04681_si_001.pdf]

Supporting Information

miRNAs-Guided Imaging and Photodynamic Therapy Treatment of Cancer Cells Using Zn(II)-Protoporphyrin IX-Loaded Metal Organic Framework Nanoparticles

*Pu Zhang[a], Yu Ouyang[a], Yang Sung Sohn[b], Michael Fadeev[a], Ola Karmi[b], Rachel Nechushtai[b], Ilan Stein[c], Eli Pikarsky[c] and Itamar Willner[a]**

[a] Institute of Chemistry, Center for Nanoscience and Nanotechnology, The Hebrew University of Jerusalem, Jerusalem 91904, Israel.

[b] Institute of Life Science, The Hebrew University of Jerusalem, Jerusalem 91904, Israel.

[c] The Lautenberg Center for Immunology and Cancer Research, IMRIC, The Hebrew University of Jerusalem, Jerusalem 91904, Israel.

E-mail: itamar.willner@mail.huji.ac.il

Experimental Procedures

Materials and Instruments. Zn(II)-protoporphyrin IX (Zn(II)-PPIX), 2-[4-(2-Hydroxyethyl)piperazin-1-yl]ethanesulfonic acid sodium salt (HEPES), disodium hydrogen phosphate (Na_2HPO_4), sodium dihydrogen phosphate (NaH_2PO_4), sodium chloride, magnesium chloride, Rhodamine 6G, N, N, N', N'-tetramethy-lethylenediamine (TEMED), acrylamide solution (40%), ammonium persulphate, dimethylformamide (DMF), zirconium oxychloride ($ZrOCl_2$) and 2-aminoterephthalic acid, 1,3-diphenylisobenzofuran (DPBF) were bought from Sigma-Aldrich. DNA oligonucleotides were synthesized and purified at Integrated DNA Technologies Inc. (Coralville, IA). Di(acetoxymethyl ester)-6-carboxy-2',7'-dichlorodihydrofluorescein diacetate (CDCHF-DA), and SYBR Gold nucleic acid gel stain was purchased from Invitrogen. Ultrapure water from NANOpure Diamond (Barnstead) source was applied throughout the whole experiments.

A Magellan XHR 400L scanning electron microscope (SEM) was employed to characterize the nanomaterials. Fluorescence spectra was measured with a Cary Eclipse Fluorometer (Varian Inc.). The excitation of both Zn(II)-PPIX and Rhodamine 6G were excited at 420 nm. The concentrations of DNA oligonucleotides were monitored using a UV-2401PC (SHIMADAZU) spectrophotometer. The gel experiment was run on a Hoefer SE 600 electrophoresis unit.

The sequences of all nucleic acids used in this paper are composed of: (from 5' to 3')

miRNA-21: UAGCUUAUCAGACUGAUGUUGA

miRNA-221: AGCUACAUUGUCUGCUGGGUUUC

miRNA-145: GUCCAGUUUUCCCAGGAAUCCCU

H_a:AGGGCGGGTGGGACTGATGTTGTTGGAGAATTGTCAACATCAGTCTGATAAG CTA

H_b:TGGTCAATTCTCCAACAACATCTAGCTTATCAGACTGATGTTGTTGGGTAGGG

CGGG

H_c:AGGGCGGGTGGGGCTGGGTTTCTTGGAGAATTGTGAAACCCAGCAGACAATG TAGCTTGGGT

H_d:TGGTCAATTCTCCAAGAAACCCTAGCTACATTGTCTGCTGGGTTTCTTGGGTA GGGCGGG

Pretreatment of hairpins. All four hairpins were pretreated to generate hairpin structure as follows (take H_a as an example): 2 μM of H_a in HEPES buffer (10 mM, 20 mM $MgCl_2$, pH = 7.2) was annealed at 95 ^oC for 5 min, subsequently, cooled down to 4 ^oC, and allowed to equilibrate at 25 ^oC for 2 hours, yielding hairpin H_a. H_b, H_c, and H_c were synthesized by following the same procedure.

Synthesis of NMOFs. The preparation of NMOFs was according to the reported method. First, 54 mg of 2-aminoterephthalic acid and 21 mg of $ZrOCl_2$ were mixed together in DMF (4 mL). Then, 2 mL of acetic acid were added to the mixture that was heated in an oven at 90 °C for 18 hours. After that, the resulting NMOFs were centrifuged and washed with DMF, triethylamine/ethanol (1:20, V/V), and ethanol, respectively.

The Zn(II)-PPIX loading and hairpins gating of NMOFs. To load Zn(II)-PPIX, 1 mg of NMOFs were shake with Zn(II)-PPIX (30 μL, 0.1 mM) for 12 h in 1 mL water. The loaded NMOFs were then transferred to a buffer solution and hybridized with respective hairpins (H_a/H_b or H_c/H_d), resulting in the locked state of the NMOFs encapsulated the load. 12 hours later, the NMOFs were washed several times to remove the unloaded load. The hairpins-locked Zn(II)-PPIX-loaded NMOFs were kept at 4 ^oC for further use.

miRNA-21-responsive H_a/H_b-gated Zn(II)-PPIX-loaded NMOFs and the release of the Zn(II)-PPIX. The miRNA-21-responsive H_a/H_b-gated Zn(II)-PPIX-loaded NMOFs, 0.1 mg,

were subjected to 1 mL respective buffer solutions (PBS or control buffer, HEPES buffer). At appropriate time intervals, samples of the mixture are centrifuged to precipitate the NMOFs (10 000 rpm for 2 minutes). Different concentrations of miRNA-21 and 50 mM K^+ were added to the supernatant solution, and incubated at room temperature for 3 h to generate the Zn(II)-PPIX/G-quadruplex photosensitizer chains. The fluorescence of the chains in the supernatant solution was measured using a Cary Eclipse Fluorescence Spectro- photometer (Varian Inc.).

miRNA-221-responsive H_c/H_d-gated Zn(II)-PPIX-loaded NMOFs and the release of the Zn(II)-PPIX. The miRNA-221-responsive H_c/H_d-gated Zn(II)-PPIX-loaded NMOFs, 0.1 mg, were subjected to 1 mL respective buffer solutions (PBS or control buffer, HEPES buffer). At appropriate time intervals, samples of the mixture are centrifuged to precipitate the NMOFs (10 000 rpm for 2 minutes). Different concentrations of miRNA-221 and 50 mM K^+ were added to the supernatant solution, and incubated at room temperature for 3 h to generate the Zn(II)-PPIX/G-quadruplex photosensitizer chains. The fluorescence of the chains in the supernatant solution was measured using a Cary Eclipse Fluorescence Spectro- photometer (Varian Inc.).

Cell culture. Epithelial breast cells (MCF-10A) were maintained in complete growth medium consisting of 1:1 mixture of Dulbecco's modified Eagle's medium and Ham's F12 medium supplemented with horse serum (5%), epidermal growth factor (20 ng/mL), cholera toxin (CT, 0.1 µg/mg), insulin (10 µg/mL), hydrocortisone (500 ng/mL), and penicillin/streptomycin (1 unit/mL). Human breast cancer cells (MDA-MB-231) were grown in RPMI-1640 medium supplemented with 10% FCS, L-glutamine, and antibiotics (Biological Industries). Human ovarian cancer cells (OVCAR-3) were grown in RPMI-1640 medium supplemented with 10% FCS, L-glutamine, bovine insulin (0.01mg/ml), and penicillin/streptomycin (1 unit/mL). Cells were plated one day prior to the experiment on 96-well plates for cell viabilities or on µ-slide 4 well glass bottom (ibidi) for confocal microscopy.

Confocal microscopy measurements. Cells, 2×10^5 were planted in μ-slide 4 well glass bottom on one day prior to the experiment. Cells were incubated with miRNA-21-responsive Zn(II)-PPIX-loaded H_a/H_b-locked NMOFs, 60 μg/mL, or miRNA-221-responsive H_c/H_d-gated Zn(II)-PPIX-loaded NMOFs, 60 μg/mL, for 6 hours and then washed with DMEM-HEPES twice, and exposed to visible light irradiation, λ= 532 nm for 12 minutes, 30 mW/cm^2. Red fluorescence in cells was monitored with the confocal microscopy (the Olympus FV3000 confocal laser-scanning microscope) and all images were analyzed with image J.

ROS production. ROS production in cancer cells was determined by incubating cells, 2×10^5, loaded with miRNA-21-responsive Zn(II)-PPIX-loaded H_a/H_b-locked NMOFs, 60 μg/mL, or miRNA-221-responsive H_c/H_d-gated Zn(II)-PPIX-loaded NMOFs, 60 μg/mL, at 37°C with 10 μM di(acetoxymethyl ester)-6-carboxy-2',7'-dichlorodihydrofluorescein diacetate (CDCHF-DA) in HEPES-buffered saline (HBS) supplemented with 10 mM glucose after the exposure of cells to the visible light (λ= 532 nm for 12 minutes, 40 mW/cm^2). This non-fluorescent molecule is readily converted to a green-fluorescent form when the acetate groups are removed by intracellular esterases and oxidation by the activity of ROS within the cells. The conversion of the non-fluorescent indicator to the green fluorescent indicator was measured on line for 1h at 37°C under the confocal microscopy (the Olympus FV3000 confocal laser-scanning microscope) (λ_{ex} = 488 nm; λ_{em} = 517 nm) and all images were analyzed with image J.

Cell viability experiments. Cell viabilities were assayed after incubation of miRNA-21-responsive Zn(II)-PPIX-loaded H_a/H_b-locked NMOFs or miRNA-221-responsive H_c/H_d-gated Zn(II)-PPIX-loaded NMOFs in MCF-10A, MDA-MB-231, OVCAR-3 cells planted at a density of 1.2×10^4 cells per well in 96-well plates. After 6 hours incubation with the respective NMOFs, 100 μg/mL, cells were washed intensively with growth medium and exposed to visible light irradiation, λ= 532 nm for 12 minutes, 40 mW/cm^2. After the exposure, cells were

further incubated with the full cell medium for 3 days. The cell viabilities were determined after 3 days with the fluorescent redox probe, Presto-Blue. The fluorescence of Presto-Blue was recorded on a plate-reader (Tecan Safire) after 1 h of incubation at 37 °C (λ_{ex} = 560 nm; λ_{em} = 590 nm).

***In vivo* study of the miRNA-21-responsive H_a/H_b-gated Zn(II)-PPIX-loaded NMOFs**

Xenograft tumor of MDA-MB-231 breast epithelial cells were followed for the growth rate and volumes in NOD-SCID mice (NS-19-15989-5) *in vivo* study. The study was approved by the Authority for Biological and Biomedical Models at the Hebrew University. 6×10^6 of MDA-MB-231 cells were injected subcutaneously to the flack of each mouse. Tumor mass was generated after 7 days in an estimated volume of 80-100 mm^3, that enabled intra-tumoral (IT) (2-3 times/ week) in total 7 injections. The female mice were divided to three groups: a saline injected control group, non-loaded NMOFs as a second control group, and the miRNA-21-responsive H_a/H_b-gated Zn(II)-PPIX-loaded NMOFs was injected to the third group which was subjected to PDT illumination with light (λ =532nm) for 15 minutes, 40 mW/cm^2. (NP+L, black lines). The injections were done in amount of 50 µg of NP for each mouse, to get NP concentration of 3.2 nmoles/ mouse. NMOFs were prepared in a final concentration of 1 mg/mL, each injection was 50 µL of either the NP or the saline. Tumor was measured every 3 days before following injection to evaluate the width and the height, then tumor volume (mm^3) was calculated using the equation of $(\text{Width}^2\times\text{Height})/2$. The tumors' growth rate was evaluated by calculating the volume for each measurement of each tumor divided by the specific tumor's starting volume value. Toxicity of the treatment was evaluated by the mice weight change (g) that was measured once a week. All results were presented as mean $\pm$ SEM.

Table S1. BET surface area and pore volume analysis of UiO-66-NH_2 NMOFs and Zn(II)-PPIX-loaded UiO-66-NH_2.

sample	BET surface area	pore volume
**UiO-66-NH_2**	1641.750 m^2/g	0.901 cc/g
**Zn(II)-PPIX-loaded UiO-66-NH_2**	1167.953 m^2/g	0.647 cc/g

These results are consistent with the binding of Zn(II)-PPIX to the pores.

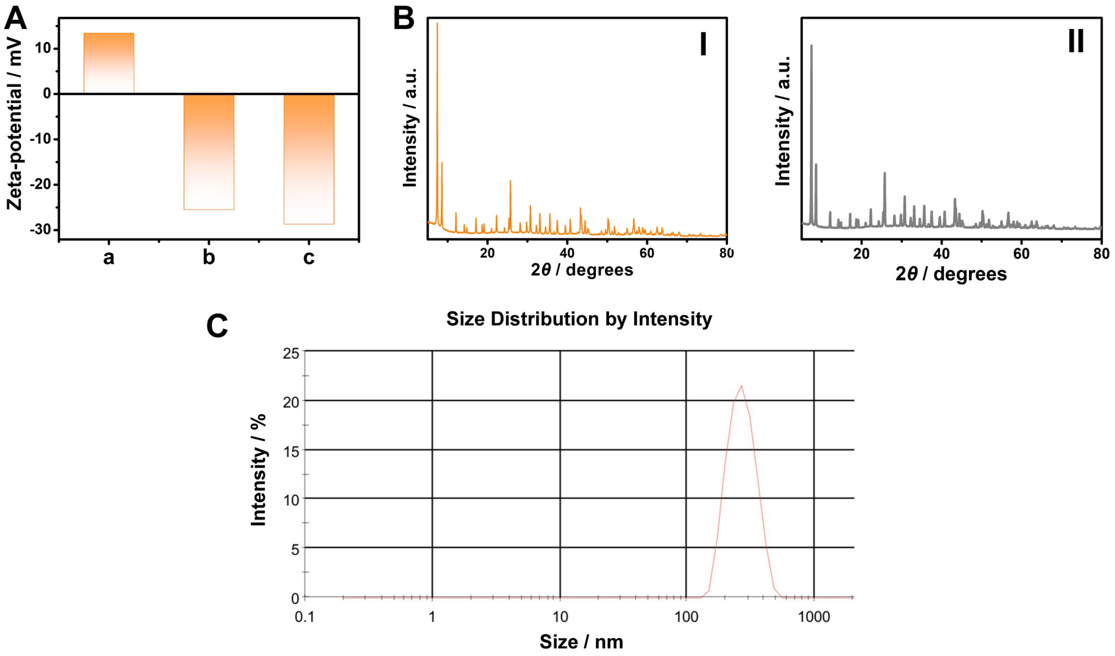

Figure S1 (A) The zeta-potentials of NMOFs. Panel a-NH_2-modified UiO-66 MOFs, UiO-66-NH_2 NMOFs; Panel b-DNA hairpins H_a and H_b-functioalized UiO-66-NH_2 NMOFs; Panel c-Zn(II)-PPIX-loaded hairpins H_a and H_b-functioalized UiO-66-NH_2 NMOFs. (B) XRD pattern before (panel I) and after modification with the hairpins H_a/H_b and loaded with Zn(II)-PPIX (panel II) of the UiO-66-NH_2 NMOFs. (C) Dynamic light scattering spectrum of the UiO-66-NH_2 NMOFs.

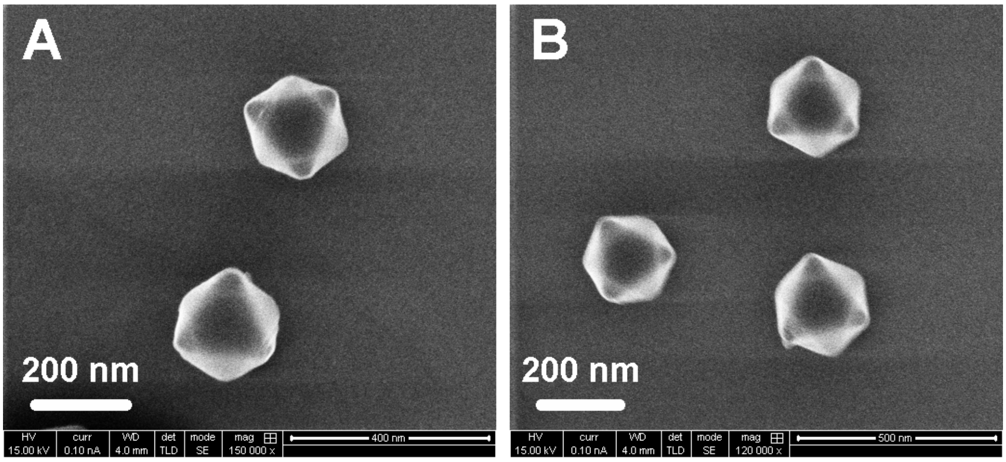

Figure S2 SEM images of (A) Zn(II)-PPIX-loaded NMOFs and (B) Zn(II)-PPIX-loaded hairpins-gated NMOFs.

These results demonstrated that the morphology of the UiO-66-NH_2 NMOFs is unchanged upon loading the particles with Zn(II)-PPIX and the gating of the loaded NMOFs with H_a and H_b.

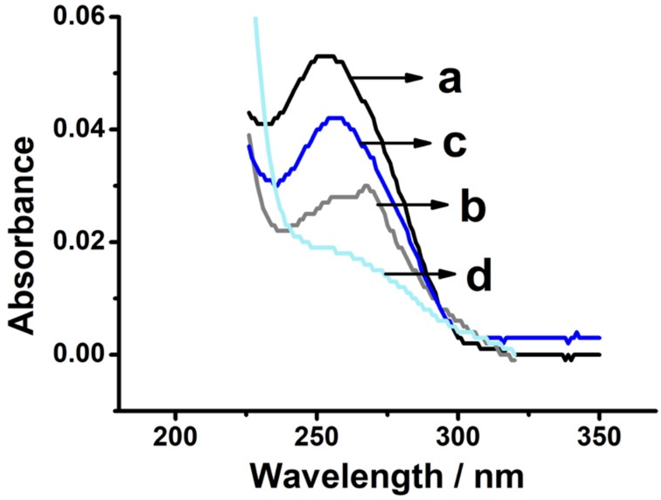

Figure S3 Evaluation of the loading of hairpins H_a and H_b associated with the NMOFs. 0.03 mg of NMOFs were introduced into a solution of 200 µL that contained 2 nmols of DNA. The absorption spectrum of the solution was recorded prior to the addition of the NMOFs. After reaction of the NMOFs with DNA hairpins, the NMOFs were precipitated and the absorption spectrum of the supernatant was recorded to evaluated the concentration of unreacted DNA hairpins. The NMOFs were washed twice with water and the spectra of the washing solution were recorded: (a) The spectrum of the first washing solution by unsubstituted teterphthalic-based UiO-66 NMOFs; (b) The spectrum of the second washing solution by UiO-66 NMOFs; (c) The spectrum of the first washing solution by amine-terphtalic acid ligand-based UiO-66-NH_2 NMOFs; (d) The spectrum of the second washing solution by UiO-66-NH_2. The concentrations of hairpins in the rinsing solution were added to the primary concentrations of non-reacted hairpins and the total concentration of residual hairpins subtracted from the initial concentration of hairpins, reacted with the NMOFs to quantitatively evaluate the loading of hairpins on the NMOFs. Using this procedure, the loading of hairpins corresponded to 15 nmols per mg of UiO-66 NMOFs and 32 nmols per mg of UiO-66-NH_2 NMOFs. It should be noted that the amine-terphtalic acid ligand was selected to bridge Zr^{4+}-ions and preferred over the unsubstituted teterphthalic ligand since we find that the loading of H_a and H_b on UiO-66-NH2 NMOFs is *ca.* 2-fold higher as compared to unsubstituted UiO-66 NMOFs. Presumably, the positive charge of amine functionalities enhances the binding affinities of negatively charged Ha/Hb to the UiO-66-NH2 NMOFs.

To further support the selective association of Zn(II)-PPIX with the K^+-stabilized G-quadruplex, the circular dichroism (CD) spectra of Zn(II)-PPIX in the presence of a random nucleic acid strand or a G-rich nucleic acid strand, in the absence of K^+-ions were recorded in the spectral region of Zn(II)-PPIX. Figure S3, curve (a) shows that subjection Zn(II)-PPIX to the G-rich strand, in the absence of K^+-ions does not show any CD response for Zn(II)-PPIX, indicating no interaction of the chromophore with the nucleic acid. In the presence of K^+-ions, subjecting of Zn(II)-PPIX to the G-rich strand yield a clear CD spectrum of Zn(II)-PPIX centered at λ=410 nm, curve (b), indicating that the Zn(II)-PPIX interacts with the K^+-stabilized G-quadruplex.

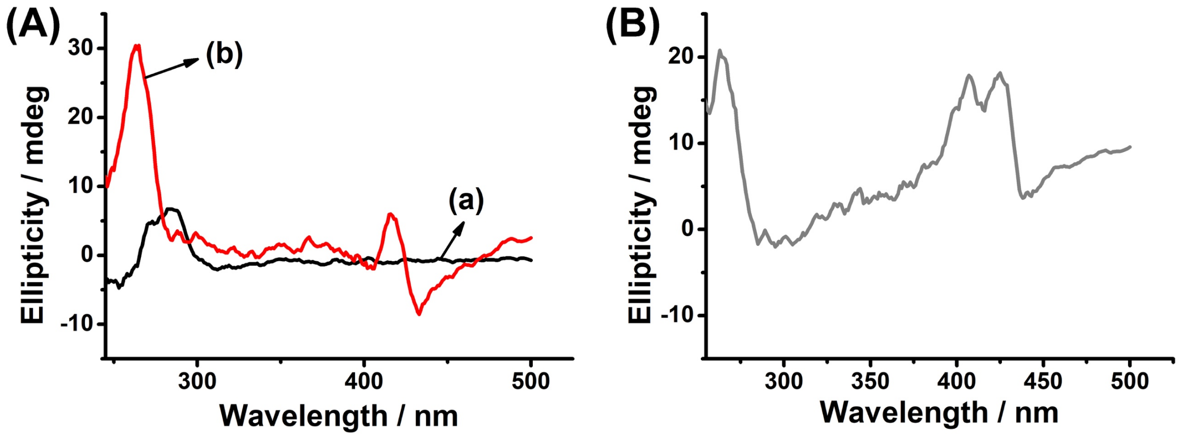

Figure S4 (A) Circular dichroism signatures of (a) Zn(II)-PPIX in the presence of a random nucleic acid strand or a G-rich nucleic acid strand, in the absence of K^+-ions. (b) In the presence of K^+-ions, subjecting of Zn(II)-PPIX to the G-rich strand. (B) Circular dichroism signatures of subjecting Zn(II)-PPIX to the G-rich strand, in the presence of K^+-ions and 10% fetal bovine serum (FBS).

The time-dependent fluorescence decay of Zn(II)-PPIX in the presence of the G-rich strand in the absence of K^+-ions is displayed in Figure S4, curve (a). A low amplitude fluorescence transient is observed. Upon the addition of K^+-ions, 50 mM, the fluorescence signal is subsequently enhanced (*ca.* 4-fold amplitude fluorescence), curve (b). These results are consistent with the enhanced fluorescence of Zn(II)-PPIX associated with the K^+-stabilized G-quadruplex.

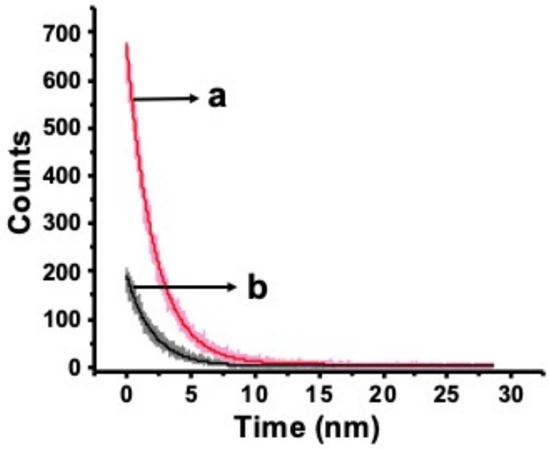

Figure S5 Time-dependent fluorescence decay of Zn(II)-PPIX in the presence of the G-rich strand in the absence of K^+-ions, curve (a), and in the presence of K^+-ions, curve (b).

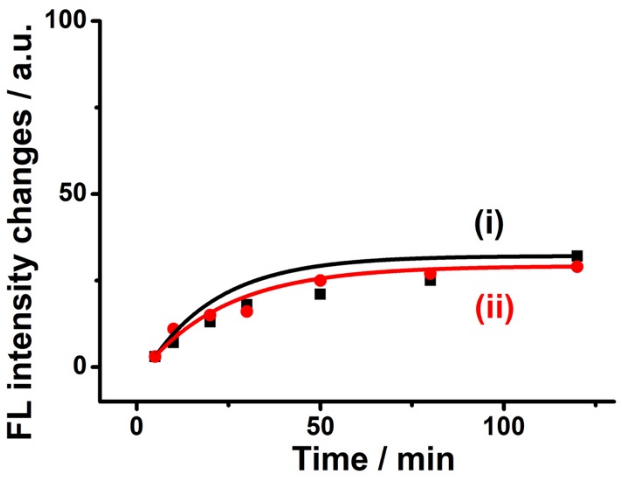

Figure S6 Time-dependent release of Zn(II)-PPIX from the miRNA-21-responsive Zn(II)-PPIX-loaded H_a/H_b-locked NMOFs: (i) In the presence of 50 mM K^+-ions, 10 mM of PBS and in the absence of miRNA; (ii) In the presence of 200 nM miRNA-21;10 mM of PBS and in the absence of K^+-ions. These control experiments revealed that in the absence of miRNA or in the absence of added K^+-ions, only minute fluorescence changes are observed, since the HCR process is prohibited, yet the low fluorescent Zn(II)-PPIX is released.

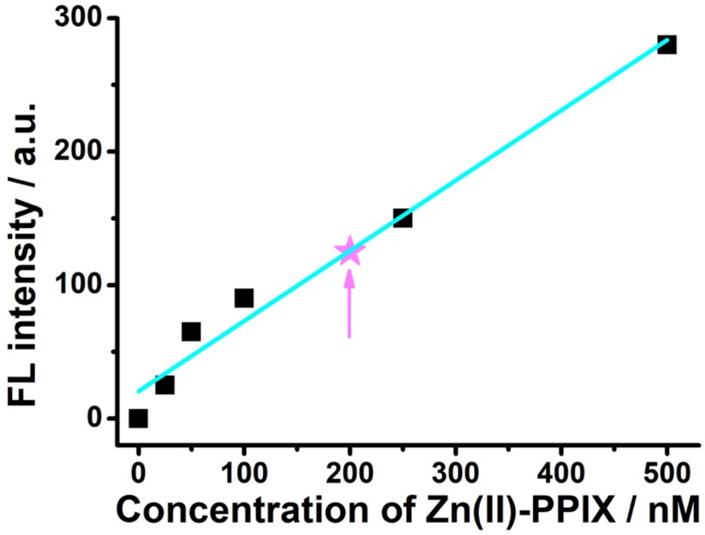

Figure S7 The calibration curve corresponding to the fluorescence intensities as a function of the concentration of Zn(II)-PPIX. The pink point is the evaluation of the loading of Zn(II)-PPIX on the NMOFs. 0.1 mg of NMOFs were introduced in 1mL of a 1 μM Zn(II)-PPIX solution. The mixture was stirred for 12 hours. Afterwards, the NMOFs were precipitated and the fluorescence spectrum of the supernatant solution was recorded, the pink point, and using the calibration curve, the loading of Zn(II)-PPIX corresponded to 80 nmols per 1mg of NMOFs.

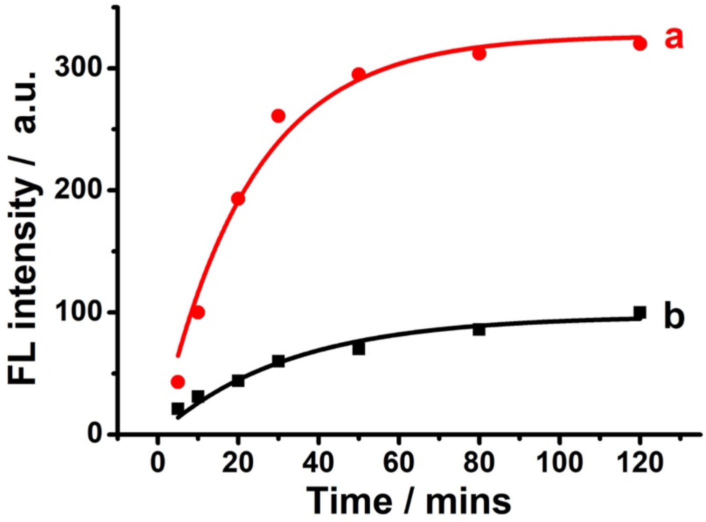

Figure S8 Time-dependent release of Rhodamine 6G from the Rhodamine 6G-loaded hairpins-locked NMOFs in the presence of curve (a), PBS; curve (b), HEPES buffer. The effective release of Rhodamine 6G from NMOFs treated with PBS was attributed to the unlocking of the hairpin gates through the separation of phosphate bonds on DNA and Zr^{4+} sites on NMOFs.

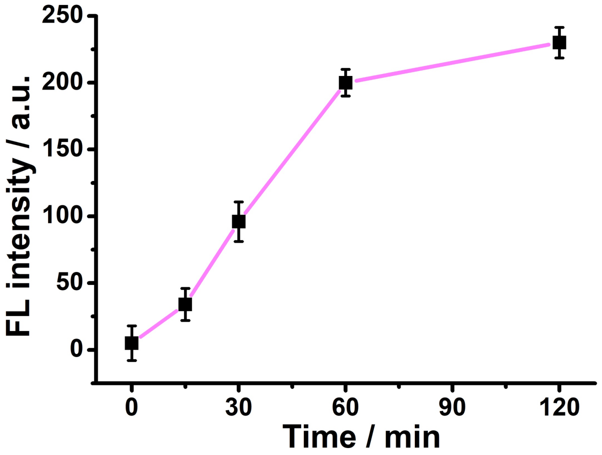

Figure S9 Time-dependent fluorescence intensities of Zn(II)-PPIX/G-quadruplex chains generated at different time-intervals of the HCR process.

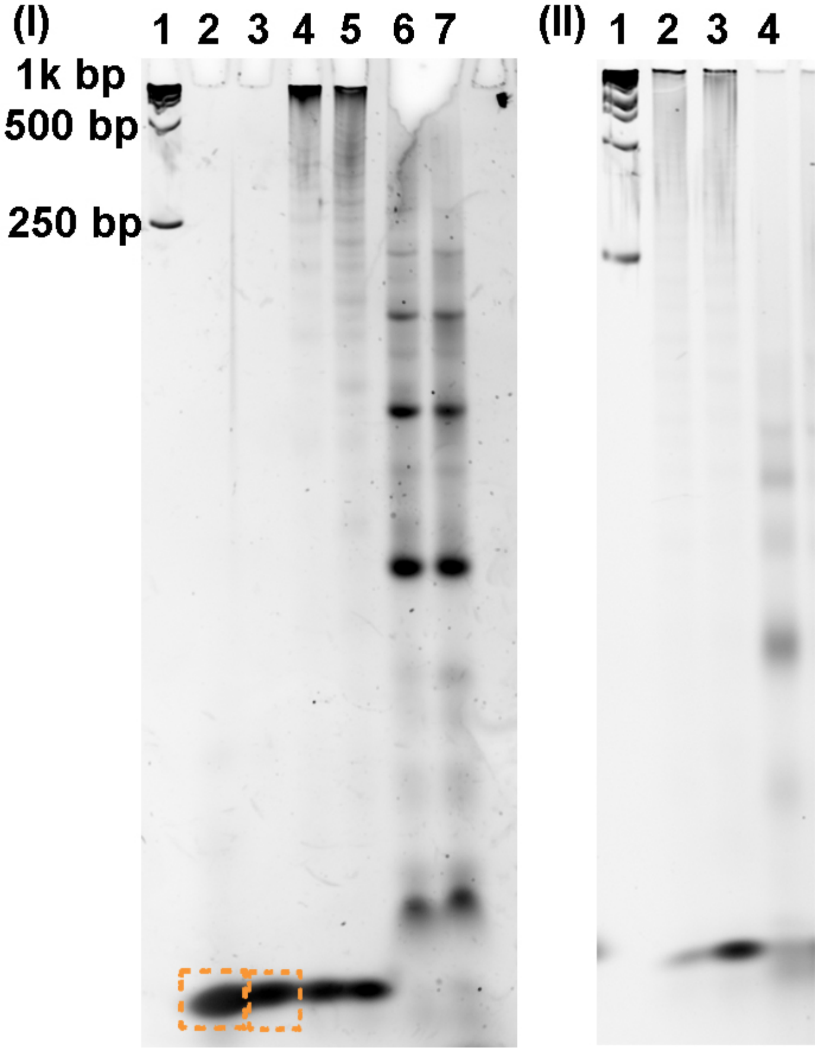

Figure S10 Electrophoretic separation following the assembly of HCR polymers by hairpins H_a and H_b, and effect of miRNA-21 concentration on HCR amplification. Electrophoresis on 12% PAGE gel (70 V, 20 h): Lane 1-DNA ladder; Lane 2-H_a; Lane 3- H_a + H_b; Lane 4-H_a + H_b, in the presence of miRNA-21, 100 nM; Lane 5-H_a + H_b, in the presence of miRNA-21, 200 nM; Lane 6-H_a + H_b, in the presence of miRNA-21, 1 μM; Lane 7-H_a + H_b, in the presence of miRNA-21, 2 μM. The miRNA-21-responsive Zn(II)-PPIX-loaded H_a/H_b-locked NMOFs (0.1 mg) were precipitated after reaction with PBS, and the supernatant was treated with different concentrations of miRNA-21. (II) Electrophoresis on 12% PAGE gel (70 V, 22 h): Lane 1-DNA ladder; Lane 2-H_a + H_b, in the presence of miRNA-21, 100 nM; Lane 3-H_a + H_b, in the presence of miRNA-21, 200 nM; Lane 4-H_a + H_b, in the presence of miRNA-21, 1 μM; The miRNA-21-responsive Zn(II)-PPIX-loaded H_a/H_b-locked NMOFs (0.1 mg) were precipitated after reaction with PBS, and the supernatant was treated with different concentrations of miRNA-21 in the presence of 10% fetal bovine serum (FBS).

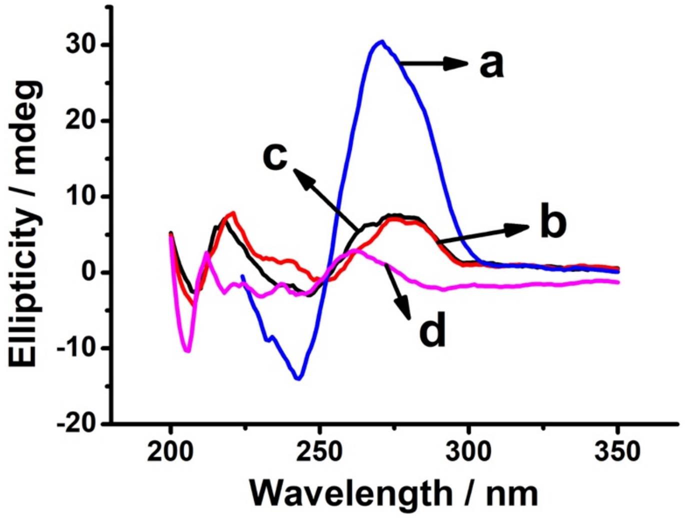

Figure S11 The miRNA-21-responsive Zn(II)-PPIX-loaded H_a/H_b-locked NMOFs (0.1 mg) were precipitated after reaction with PBS, and the circular dichroism signatures of the supernatant was recorded to evaluate the formation of G-quadruplex and control systems. Circular dichroism signatures of (a) residue solutions containing H_a and H_b, in the presence of 50 mM K^+-ions and 500 nM miRNA-21. (b) residue solutions containing H_a and H_b, in the presence of 50 mM K^+-ions and in the absence of miRNA-21. (c) miRNA-21 (1 μM) in PBS solution. (d) H_a (1 μM) in PBS solution.

Probing the effect of phosphate-ions-stimulated unlock of the NMOFs on the kinetics of diffusional release of Zn(II)-PPIX from the NMOFs and its possible effect on the resulting miRNA-21 triggered HCR formation of the Zn(II)-PPIX-loaded G-quadruplex wires.

The unlocking of the NMOFs includes phosphate-ions displacement of the hairpins H_a and H_b and the parallel diffusional release of Zn(II)-PPIX. The release of the hairpins H_a and H_b could immediately affect the miRNA-21 triggered HCR wires function and the possible diffusional constrains on the release of the Zn(II)-PPIX could affect the efficiency (rate) of formation of the final Zn(II)-PPIX/G-quadruplex-loaded wires. To address this issue, several control experiments were performed. In one experiment, Figure S12 (A), the unloaded NMOFs were modified with hairpins H_a and H_b functionalized with the FAM fluorophore. The phosphate ions release rate of the hairpins was examined, in the absence of miRNA-21, and it is displayed in Figure S12 (B), curve (a). For comparison, the rate of the miRNA-21 triggered formation of the Zn(II)-PPIX/G-quadruplex-loaded wires is displayed in Figure S12 (B), curve (b). The rates of the two independent processes are almost similar. These results imply that the formation of the miRNA-triggered formation of the Zn(II)-PPIX/G-quadruplex wires is neither controlled by the HCR process itself by the diffusional release of Zn(II)-PPIX (that is fast). **The kinetics of the entire process is dictated by the displacement of the hairpins by phosphate ions.**

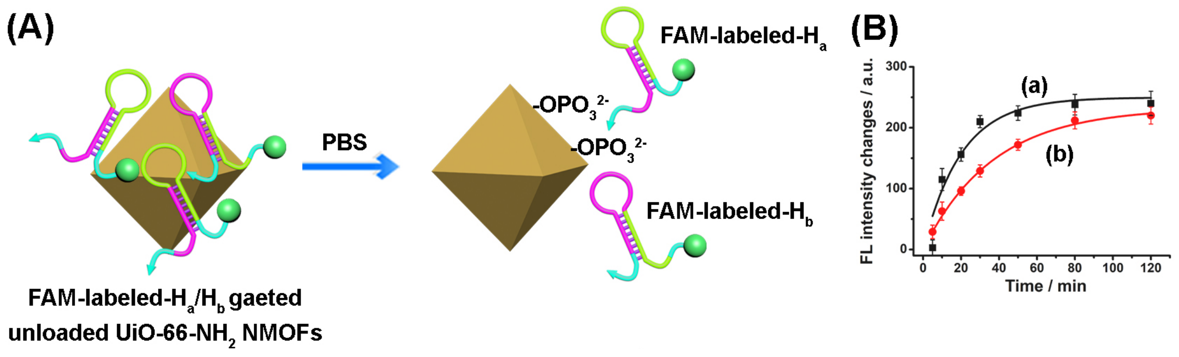

Figure S12 (A) Scheme for the unloaded UiO-66-NH_2 NMOFs and their gating by FAM fluorophore-modified hairpins H_a and H_b. The bound hairpins are displaced by phosphate-ions, resulting in the release of FAM fluorophore-modified hairpins H_a/H_b. (B) (a) Time-dependent fluorescence changes of FAM fluorophore generated by FAM-labeled H_a/H_b-locked unloaded NMOFs treated with 100 μL of PBS, 10 mM. (b) Time-dependent fluorescence changes of Zn(II)-PPIX/G-quadruplex chains generated by miRNA-21-responsive Zn(II)-PPIX-loaded H_a/H_b-locked NMOFs treated with PBS. 0.1 mg NMOFs are treated with 100 μL of PBS, 10 mM, miRNA-21, 200 nM, and K^+-ions, 50 mM.

In a second control experiment, Figure S13 (A), we fascinated Zn(II)-PPIX-loaded NMOFs that are locked by foreign H_x H_y hairpins and subjected these functionalized NMOFs to phosphate ions and a solution that include a pre-prepared K^+-stabilized G-quadruplex. Figure S13 (B), curve (a) presents the rate of formation of the Zn(II)-PPIX/G-quadruplex as a result of phosphate ions displacement of hairpins H_x H_y and the release of the Zn(II)-PPIX. For comparison, Figure S13 (B), curve (b), depicts the rate of the miRNA-21 triggered HCR formation of the Zn(II)-PPIX/G-quadruplex wires upon unlocking the H_a/H_b hairpins. The rates of the two-independent process are almost identical. These results reconfirmed that the release of the hairpins locks is the rate-limiting step in the formation of the Zn(II)-PPIX/G-quadruplex wires. **That is, the miRNA-driven process or the diffusional release of Zn(II)-PPIX are not rate-limiting in the formation of the active Zn(II)-PPIX/G-quadruplex wires.**

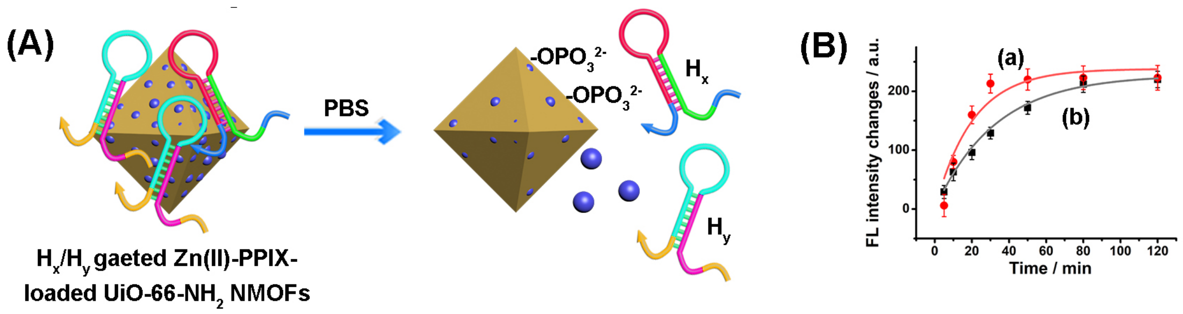

Figure S13 (A) Scheme for the loading of NMOFs with Zn(II)-PPIX photosensitizer and their gating by hairpins H_x and H_y. The bound hairpins are displaced by phosphate-ions, resulting in the release of hairpins H_x/H_y. (B) (a) Time-dependent fluorescence changes of Zn(II)-PPIX/G-quadruplex chains generated by Zn(II)-PPIX-loaded H_x/H_y-locked NMOFs treated with 100

μL of PBS, 10 mM, including pre-prepared K^+-stabilized G-quadruplex. (b) Time-dependent fluorescence changes of Zn(II)-PPIX/G-quadruplex chains generated by miRNA-21-responsive Zn(II)-PPIX-loaded H_a/H_b-locked NMOFs treated with PBS. 0.1 mg NMOFs are treated with 100 μL of PBS, 10 mM, miRNA-21, 200 nM, and K^+-ions, 50 mM.

miRNA-221-guided the formation of Zn(II)-PPIX/G-quadruplex wires from the miRNA-221-responsive H_c/H_d-gated Zn(II)-PPIX-loaded NMOFs

The result in Figure 2 (F) suggests, however, that appropriate engineering of hairpins recognizing other miRNAs and the design of hairpins-modified NMOFs could yield other selective miRNAs-responsive NMOFs. Indeed, the miRNA-guided HCR-stimulated generation of fluorescent Zn(II)-PPIX/G-quadruplex chains by the miRNA-221 that acts as specific biomarker for ovarian cancer cells was demonstrated, Figure S14 and accompanying discussion. the miRNA-221-responsive Zn(II)-PPIX-loaded NMOFs were gated by the haripins H_c/H_d, where H_c included the recognition sequence for miRNA-221 in its stem domain, and H_c/H_d were engineered to allow, upon opening hairpin H_c by miRNA-221, the inter-hairpin opening and polymerization of H_c/H_d to yield the G-quadruplex biopolymer wires, Figure S14 (A). In the presence of PBS, H_c and H_d are dissociated from NMOFs, leading to the release of Zn(II)-PPIX that associate to G-quadruplex units and generating highly fluorescent Zn(II)-PPIX/G-quadruplex chains. The time-dependent fluorescence changes upon the miRNA-221-guided synthesis of Zn(II)-PPIX/G-quadruplex fluorescent chains in the presence of PBS, is depicted in Figure S14 (B), curve (i). As before, the miRNA-221-responsive H_c/H_d-gated Zn(II)-PPIX-loaded NMOFs were treated with non-phosphate containing HEPES buffer, curve (ii). Effective release of Zn(II)-PPIX from PBS treated NMOFs is observed, as compared to the release of load from NMOFs treated with HEPES. In addition, treatment of the miRNA-221-responsive NMOFs with other foreign miRNAs does not lead to the generation of fluorescent Zn(II)-PPIX/G-quadruplex chains, Figure S14 (B), curve (iii): miRNA-21, curve (iv): miRNA-145. The fluorescent spectra of Zn(II)-PPIX/G-quadruplex chains generated by NMOFs treated with miRNA-221 and foreign miRNAs, in a fixed time interval of 40 mins, is shown in Figure S15. The formation of fluorescent Zn(II)-PPIX/G-quadruplex wires is controlled by the concentration of miRNA-221, and as the concentration of triggering miRNA

increase, the fluorescence of the wires is intensified, Figure S14 (C). The formation of G-quadruplex chains triggered by miRNA-221 was confirmed by electrophoretic separation, Figure S16. The miRNA-dictated generation of highly fluorescent Zn(II)-PPIX wires suggested the potential use of the systems for selective imaging and for selective PDT treatment of respective malignant cells. That is the fluorescent features of the resulting wires could be used as effective photosensitizers for the generation of ROS, acting as cytotoxic agents toward malignant cells.

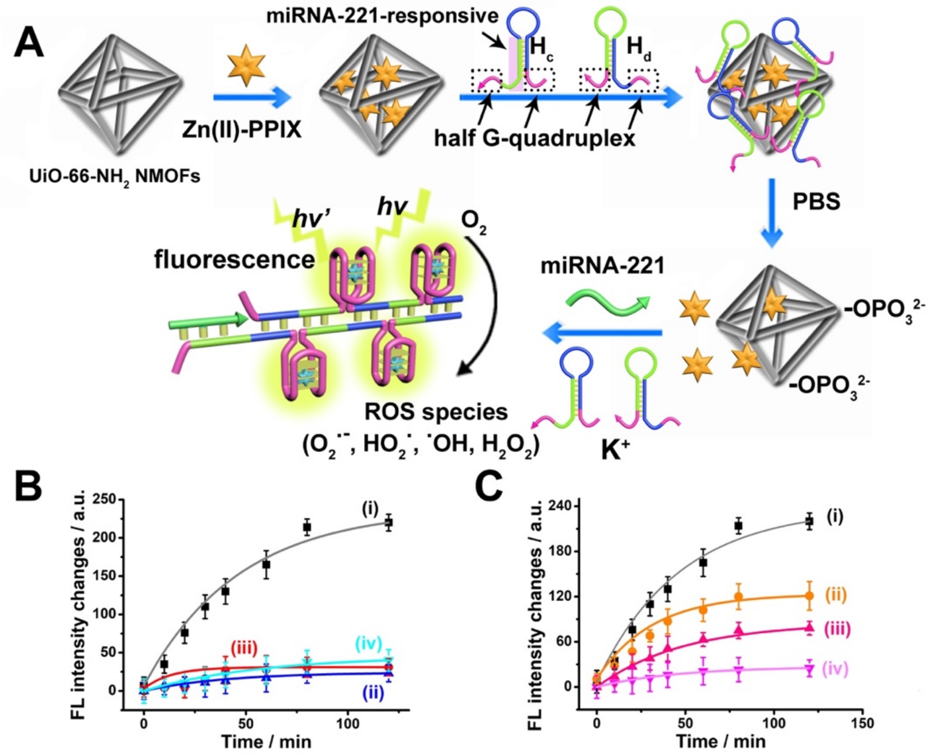

Figure S14 (A) Schematic application of the miRNA-221-responsive H_c/H_d-gated Zn(II)-PPIX-loaded NMOFs for PBS-induced release of Zn(II)-PPIX and miRNA-221-triggered HCR, generating the fluorescent Zn(II)-PPIX/G-quadruplex wires. (B) Time-dependent fluorescence changes generated upon miRNA-221-responsive H_c/H_d-gated Zn(II)-PPIX-loaded NMOFs treated with (i) PBS and miRNA-221; (ii) HEPES buffer and miRNA-221; (iii) PBS and miRNA-21; (iv) PBS and miRNA-145. 0.1 mg NMOFs are treated with 100 µL of PBS or HEPES buffer, 10 mM, in the presence of respective miRNAs, 200 nM, and K^+-ions, 50 mM. (C) Time-dependent fluorescence changes of Zn(II)-PPIX/G-quadruplex wires generated by variable concentrations of miRNA-221, (i) 200 nM; (ii) 100 nM; (iii) 50 nM; (iv) 0 nM. 0.1 mg NMOFs are treated with 100 µL of PBS, 10 mM, and K^+-ions, 50 mM. Error bars derived from N=3 experiments.

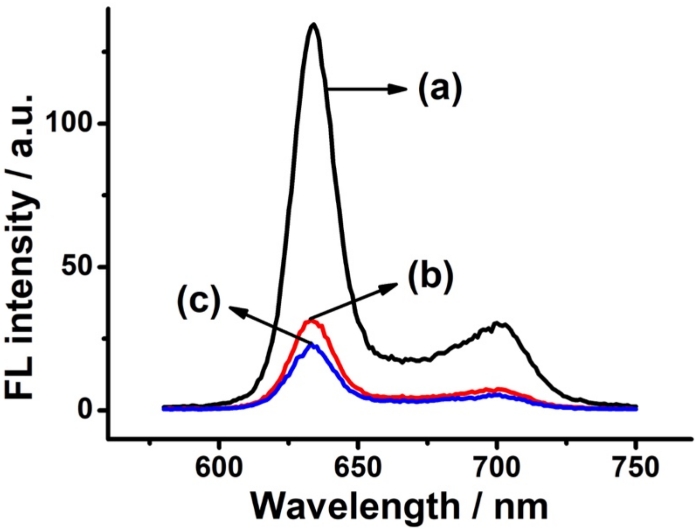

Figure S15 Fluorescence spectra of Zn(II)-PPIX released from the miRNA-21-responsive H_a/H_b-gated Zn(II)-PPIX-loaded NMOFs in the presence of: (a) miRNA-21; (b) miRNA-221; (c) miRNA-145. Spectra recorded after a time-interval of 40 minutes, concentrations of the different miRNAs corresponded to 200 nM.

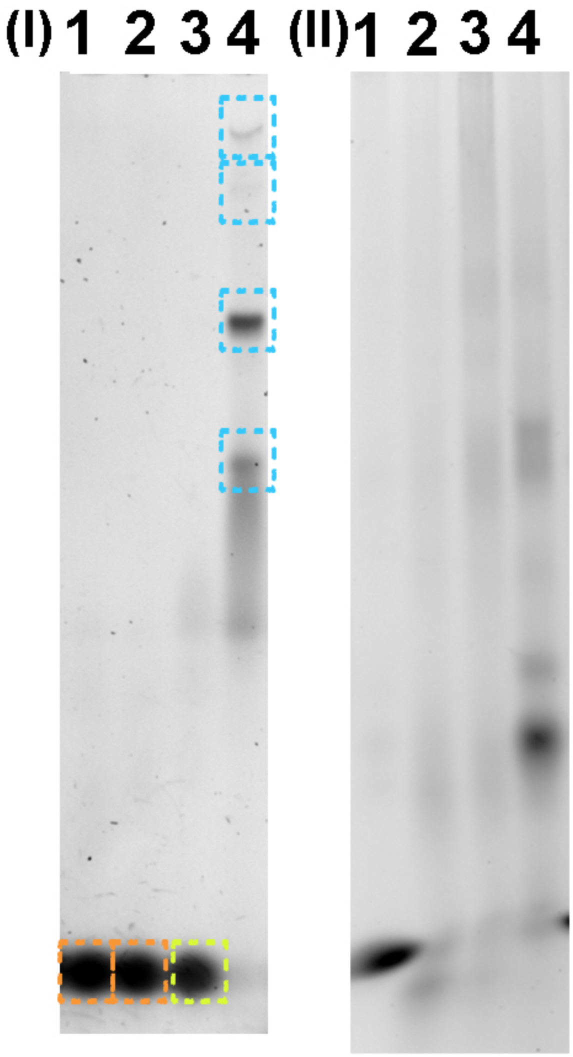

Figure S16 Electrophoretic separation following the assembly of HCR polymers by hairpins H_c and H_d and effect of miRNA-221 concentration on HCR amplification. Electrophoresis on 12% PAGE gel (70 V, 20 h): Lane 1-H_a; Lane 2-H_b; Lane 3-H_a + H_b; Lane 4-H_a + H_b, in the presence of miRNA-221, 1 μM. The miRNA-221-responsive Zn(II)-PPIX-loaded H_c/H_d-locked NMOFs (0.1 mg) were precipitated after reaction with PBS, and the supernatant was treated with 1 μM of miRNA-221. (II) Electrophoresis on 12% PAGE gel (70 V, 19 h): Lane 1-H_a + H_b; Lane 2-H_a + H_b, in the presence of miRNA-221, 100 nM. Lane 3-H_a + H_b, in the presence of miRNA-221, 200 nM. Lane 4-H_a + H_b, in the presence of miRNA-221, 1 μM. The miRNA-221-responsive Zn(II)-PPIX-loaded H_c/H_d-locked NMOFs (0.1 mg) were precipitated after reaction with PBS, and the supernatant was treated with different concentrations of miRNA-221 in the presence of 10% fetal bovine serum (FBS).

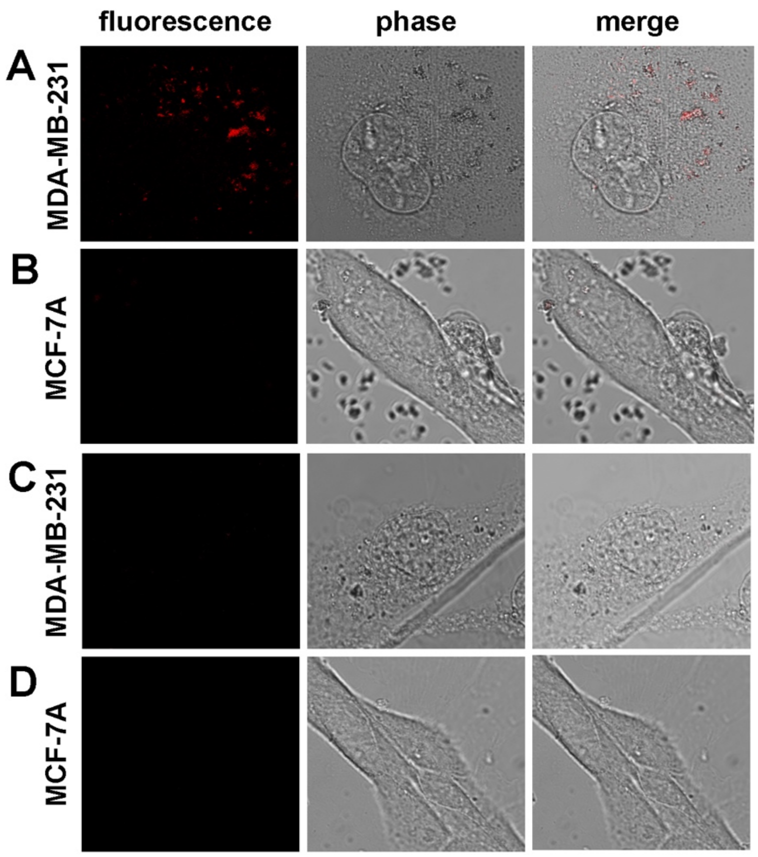

Figure S17 Confocal microscopy images of: (A) MDA-MB-231 cells treated with miRNA-21-responsive H_a/H_b-gated Zn(II)-PPIX-loaded NMOFs. (B) MCF-7A cells treated with miRNA-21-responsive H_a/H_b-gated Zn(II)-PPIX-loaded NMOFs. (C) MDA-MB-231 cells treated with naked solution containing H_a/H_b and Zn(II)-PPIX. (D) MCF-7A cells treated with naked solution containing H_a/H_b and Zn(II)-PPIX. It should be noted that the content of H_a/H_b and Zn(II)-PPIX is the same in both NMOFs carriers and naked solution. Compared to the naked hairpin/Zn(II)-PPIX solution, cellular uptake of haripin-gated Zn(II)-PPIX-loaded NMOFs was significantly enhanced in MDA-MB-231 cells, indicating that the NMOFs protect hairpins from nuclease degradation and enhance cellular uptake of the loads, facilitating the internalization *via* endocytosis pathways.

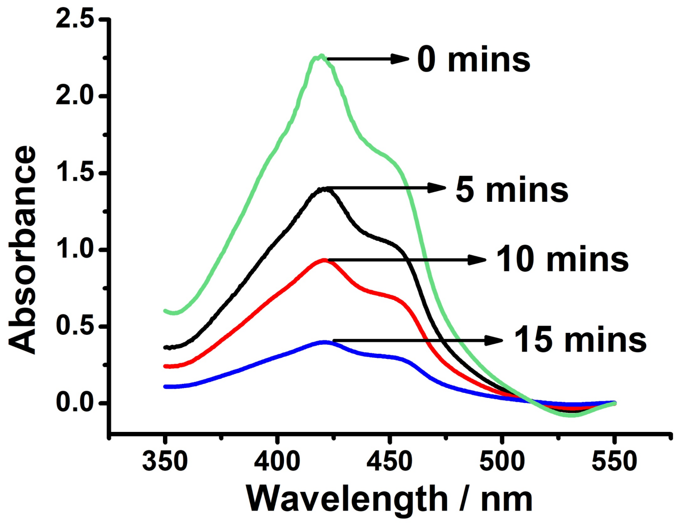

Figure S18 The photodynamic efficacy of miRNA-21-responsive Zn(II)-PPIX-loaded H_a/H_b-locked NMOFs was determined by measuring ROS generation using DPBF as an indicator. The miRNA-21-responsive Zn(II)-PPIX-loaded H_a/H_b-locked NMOFs (0.2 mg) were precipitated after reaction with PBS, and the supernatant was treated with 50 mM K^+-ions and of 500 nM miRNA-21, and then was mixed with DPBF (20 μM, in DMSO). The prepared samples were exposed to laser for different time-intervals (λ= 532 nm, 30 mW/cm^2) and the progress of the reaction was recorded in the absorbance of DPBF at 410 nm, and was monitored by UV–vis spectrometer.

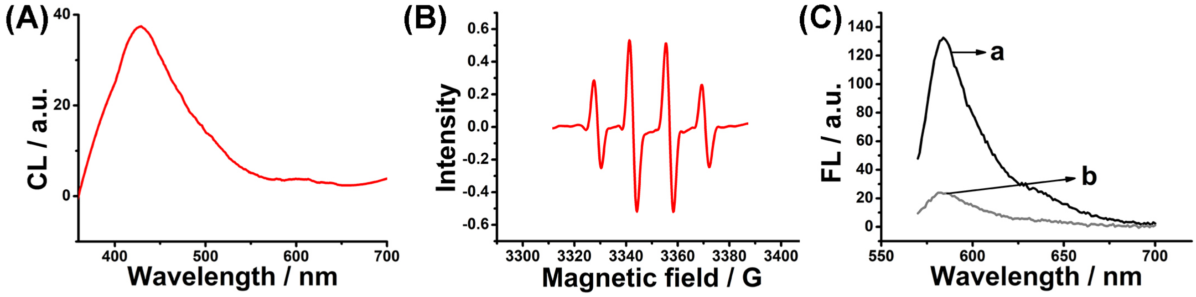

Figure S19 (A) Chemiluminescence spectra upon analyzing $O_2^{\bullet-}$ by Zn(II)-PPIX/G-quadruplex photosensitizer chains generated by treating the miRNA-21-responsive H_a/H_b-gated Zn(II)-PPIX-loaded NMOFs to PBS and miRNA-21 under irradiation. (B) ESR spectrum corresponding to the •OH generated by Zn(II)-PPIX/G-quadruplex photosensitizer chains generated by treating the miRNA-21-responsive H_a/H_b-gated Zn(II)-PPIX-loaded NMOFs to PBS and miRNA-21 under irradiation. (C) Fluorescence spectra of Resorufin upon analyzing H_2O_2 generated by Zn(II)-PPIX/G-quadruplex photosensitizer chains generated by treating the miRNA-21-responsive H_a/H_b-gated Zn(II)-PPIX-loaded NMOFs to PBS and miRNA-21: curve (a) with irradiation, curve (b) without irradiation. The irradiation was under visible light, λ= 532 nm for 30 minutes, 30 mW/cm^2.
